# Time-dependent endpoints as predictors of overall survival in multiple myeloma

**DOI:** 10.1186/1471-2407-13-122

**Published:** 2013-03-16

**Authors:** Jorge Félix, Filipa Aragão, João M Almeida, Frederico JM Calado, Diana Ferreira, António BS Parreira, Ricardo Rodrigues, João FR Rijo

**Affiliations:** 1 Exigo Consultores, Av. Humberto Delgado 33, Alhos Vedros, 2860-021, Portugal; 2 Portuguese Oncology Institute–Lisboa, Lisbon, Portugal; 3 Faculty of Pharmacy, Universidade de Lisboa, Lisbon, Portugal; 4 Hospital Egas Moniz, Lisbon, Portugal

**Keywords:** Multiple myeloma, Overall survival, Survival predictors, Time-dependent endpoint, Time to progression

## Abstract

**Background:**

Supporting health care sector decisions using time-dependent endpoints (TDEs) such as time to progression (TTP), progression-free survival (PFS), and event-free survival (EFS) remains controversial. This study estimated the quantitative relationship between median TDE and median overall survival (OS) in multiple myeloma (MM) patients.

**Methods:**

Studies (excluding allogeneic transplantation) published from 1970 to 2011 were systematically searched (PubMed). The nonparametric Spearman’s rank correlation coefficient measured the association between median TDE and OS. The quantitative relationship between TDEs and OS was estimated with a two-step approach to a simultaneous Tobit model.

**Results:**

We identified 153 studies: 230 treatment arms, 22,696 patients and mean study duration of 3.8 years. Mean of median TDEs was 22.5 months and median OS was 39.1 months. Correlation coefficients of median TTP, PFS, and EFS with median OS were 0.51 (P = 0.003), 0.75 (P < 0.0001), and 0.84 (P < 0.0001), respectively. We estimate a 2.5 month (95% confidence interval, 1.7–3.2) increase in median OS for each additional month reported for median TDEs. There was no evidence that this relationship differed by type of surrogate.

**Conclusion:**

TDEs predict OS in MM patients; this relationship may be valuable in clinical trial design, drug comparisons, and economic evaluation.

## Background

Multiple myeloma (MM) is the second most common hematologic malignancy, after non-Hodgkin lymphoma [1,2]. In the US, it was estimated that over 20,500 new cases of MM and more than 10,600 deaths occurred in 2011 [3]. Despite improved survival over the past decades, MM remains an incurable disease, with research focused on finding more effective treatments [4]. Although improving overall survival (OS) has been the gold standard outcome for new anticancer treatments, large costly trials with long follow-up periods are required to document an impact on OS [5,6]. Furthermore, OS can be influenced by trial design characteristics, such as crossover and sequential treatments [7,8]. Therefore, surrogate endpoints that can be measured sooner and more frequently during the course of a clinical trial, are being used to provide an earlier indication of efficacy [9].

A surrogate endpoint is a measurement that can be substituted for the final endpoint (e.g., improvement in OS) to successfully measure the effect of an intervention [10]. Common surrogate endpoints for OS used in clinical oncology trials include: response rate; time to disease progression (TTP); progression-free survival (PFS); and event-free survival (EFS) [6]. For study conclusions to be valid, differences or changes observed in the surrogate endpoints must accurately reflect changes in the final endpoint [11]. There is ongoing debate about the utilization of these time-dependent endpoints (TDEs) as intermediate endpoints for OS in clinical trials [12,13], as well as their value to health authorities when assessing drug approvals and assessing costs of drug therapy [7,14,15].

In 1992, the US Food and Drug Administration (FDA) instituted the accelerated approval process to allow earlier marketing of drugs that treat serious, life-threatening diseases [16]. Recently, the FDA ruled that both TTP and PFS are valid and clinically relevant TDEs that can be used in the accelerated approval process for MM agents [17]. Although these endpoints are generally thought to be reliable in MM, their predictive value for OS is unknown. Our objective was to estimate a quantitative relationship between median TDEs and median OS from prospective published MM studies in order to address the question of what the expected median OS would be given the observed effect in the median TDE.

## Methods

### Search strategy and selection criteria

We systematically searched PubMed for articles published between January 1970 and February 2011 to identify experimental or observational prospective studies that assessed OS in MM using TTP, PFS, or EFS as a primary endpoint. Key search words included “myeloma” or “survival” or “progression”; see Additional file 1 for full search details. Retrospective studies were excluded, as were studies involving allogeneic transplantation, which generally target younger patients with a clinical status that differs greatly from the majority of MM patients. The TDEs considered were median TTP, PFS, and EFS. The final outcome measures considered were median OS, 12-month OS, 24-month OS, 36-month OS, or any survival data that described the proportion of patients alive or deceased and the duration of follow-up. Studies lacking surrogate endpoint outcomes or OS data were excluded; see Additional file 1 for the selection process scheme. The following variables were collected: authors, publication year, journal, study sample characteristics (period of analysis, median age, percentage of males, type and number of previous therapies), and study results (therapies used, median TDEs, median OS, 12-month OS, 24-month OS, and 36-month OS).

### Statistical methods

Using the median TDE as an independent observation to estimate median OS, with each study arm representing a single observation, presents a statistical challenge. Outcome measures such as TTP, PFS, and EFS differs on their exact definition, especially with the inclusion or exclusion of death as event [17]. Concerning the estimation of the statistical model, this points to the presence of endogeneity of the main regressor of interest (TDE) and heterogeneity of observations. In the context of linear regression, an estimation based on an instrumental variables approach may be considered, when the endogeneity of regressors is suspected [18].

In addition, the data analyzed are sourced from a literature review of studies with different study designs, patient populations, and treatments. It is possible that the variance of the unexplained share (the residuals) differs among observations and that heteroskedasticity (non-constant variance) is present. In the presence of heteroskedasticity, the instrumental variables estimator yields consistent, but inefficient, estimates of the coefficients and an inconsistent estimate of the covariance matrix [19]. One way to minimize inefficiency in the coefficient estimates is to weight each observation by the number of patients enrolled in the corresponding study arm and to use the Generalized Method of Moments (GMM) [19]. Inconsistency of the standard error estimates may be corrected by using heteroskedasticity robust estimators of the variance–covariance matrix, such as the Huber/White sandwich estimator [20,21].

The set of available exogenous variables (candidates to instruments) comprise the following: 12-months OS rate; proportion of females; median age; dummy variables characterizing patients by previous treatment; type of TDEs; and publication year.

Not all study arms included in the review reached the median OS by the last published follow-up; those that did not are censored observations. If, at the end of a given study, less than 50% of the patients at risk were alive, then the exact value of the dependent variable being modeled (median OS) is known. However, if the duration of the study is shorter than the number of months needed to have less than 50% of patients alive, then all that is known about the dependent variable is that it is higher than the study duration. This information is potentially relevant and was included in our analysis.

The estimation of the censored model with one endogenous variable (TDE) was performed by a two-step estimation process similar to the method developed by Smith and Blundell [22], with the following modifications:

(i) The regression of the endogenous variable on the excluded instruments and exogenous variables (Step 1) was weighted by the number of patients in each study arm and estimated using the GMM Cragg estimator [23]. This estimator makes use of information provided by the excluded instruments (median age, percentage of males, and classification of patients by number of previous treatments) to increase efficiency in the presence of heteroskedasticity of unknown form.

(ii) Given that the censoring point varies with the study arm, a censored normal-weighted regression with the robust option [21] was estimated in Step 2, instead of a purely Tobit model.

Following the approach described in Smith and Blundell (1986) [22], variance of the estimators’ formulas was corrected for one endogenous regressor. With such corrections Smith and Blundell demonstrate that estimates for standard errors, t-statistics, and confidence intervals (CIs) are asymptotically valid and hence applicable in the context of the present analysis.

Evidence in favor of the use of the instrumental variable approach was generated by the Durbin-Wu-Hausman test for endogeneity of the TDE regressor [24-26]. The validity of the instruments used was confirmed by both a high degree of correlation between the instruments and the endogenous regressor and the orthogonality between the endogenous regressor and the error term (Hansen J-statistic) [27]. The Breusch-Pagan test for heterogeneity [28] was used to check for the adequacy of the GMM estimator. The selection of the final functional form of the model was based on the RESET test [29], a test that checks the possible omission of relevant variables or, more specifically, validates the linearity assumption.

Alternative model specification was based on three criteria: validity of the instruments used; explanatory power of the instruments in the first-stage regression; and AIC (Akaike Information Criteria) in the second-stage (censored) regression.

The association between median TDE and median OS was quantified through Spearman’s rank correlation coefficient in a restricted subset of data consisting of trials only with simultaneously observed values for median TDE and median OS, and excluding those trials with unobserved median OS values [30,31]. This complete-case analysis is known to result in loss of accuracy and precision when the data are not missing completely at random [32], but was assumed in order to avoid data imputation methods or assumptions about the distribution of the unobserved median OS values. The analysis was performed with the Stata Statistical Software (Release 11. College Station, TX: StataCorp LP).

## Results

### Sample characteristics

Of the 1,061 studies retrieved, we included 153 studies involving 230 study arms (see Additional file 1). The primary endpoints reported were: median TTP (n = 46); median EFS (n = 76); and median PFS (n = 108). Of the 230 study arms, 163 reported median OS (67 unobserved values). The 12-, 24-, and 36-month OS rates were reported in 100%, 73%, and 70% of the study arms, respectively. Overall, the sample included 22,696 MM patients, 56.5% of whom were male (Table 1). Each arm included an average ± standard deviation of 99 ± 80 patients (range 8–345). The mean of reported median age was 61.3 ± 7.2 years (range 44–79), and the majority of arms (67.6%) represented only newly diagnosed or treatment-naïve patients. Mean of median TDEs was 22.5 ± 15.2 months (range 3–21), and the mean of median OS was 39.1 ± 18.4 months (range 8–126).

**Table 1:** Characteristics of the studies and patients included in the analysis

**Characteristic**	**(n = 22,696)**
Males,%	56.5
Age in years, mean of medians ± standard deviation^a^	61.3 ± 7.2
Multiple myeloma, no. of patients (study arms)	
Newly diagnosed	15,345 (128)
Relapsed, refractory, or advanced	5,273 (68)
Mixed or not reported^b^	2,078 (34)
Duration of the studies (years), mean ± standard deviation	3.8 ± 2.0
Mean of median time-dependent endpoint, months (range)	22.5 (3–121)
Time to progression	16.7 (4–39)
Progression-free survival	22.7 (5–121)
Event-free survival	25.7 (3–70)
Median overall survival in months, mean (range)	39.1 (8–126)

^a^Age statistics represent the mean of medians because the majority of studies reported the median rather than the mean age of the studied population.

^b^Corresponds to those studies including: newly diagnosed; relapsed, refractory, or advanced; or to those not differentiating the type of patients.

### Correlation between TDEs and OS

**Figure 1:**
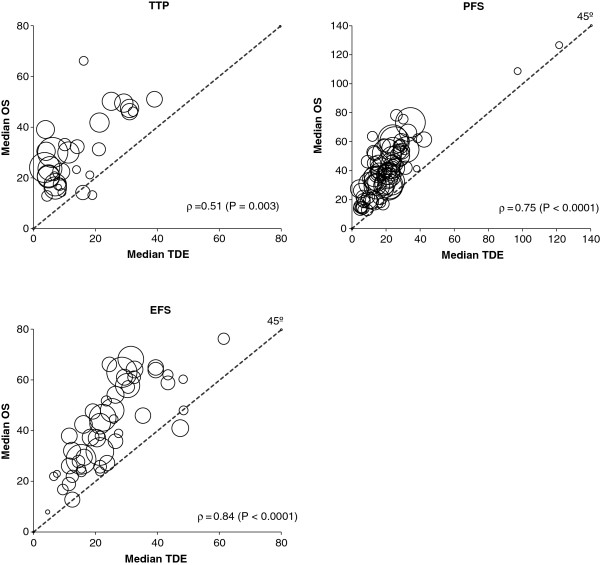
**Correlation between median values of the time-dependent endpoints (TDEs) and observed median overall survival (OS).** Each circle represents a study arm, and the surface area of the circle is proportional to the size of the arm. The nonparametric Spearman’s rank correlation coefficient (*ρ*) and its level of significance are also reported. Abbreviations: EFS, event-free survival; PFS, progression-free survival; TTP, time to progression.

The Spearman’s rank correlation coefficient of the aggregated median TDE data on median OS was 0.78 (P < 0.0001). Figure 1 shows the correlation between median values of the TDEs and median values of observed OS. The nonparametric Spearman’s rank correlation coefficient value (*ρ*) was highly significant for all TDEs, with a moderate correlation between median OS and median TTP (*ρ* = 0.51), and a strong correlation for median PFS (*ρ* = 0.75) and median EFS (*ρ* = 0.84).

### Modeling the effect of TDE on OS

Table 2 reports the regression of identified variables in median OS. Regression coefficients (*β*) correspond to the estimated effect of each variable controlled for the effect of all other variables included in the model. The 95% CIs including zero identify a non-significant effect. We found an increase of 2.45 months (95% CI, 1.71–3.20) in the reported median OS for each additional month in the observed median TDE (Table 2). This estimate was obtained adjusting for differences in the study demographics, patient type, surrogate endpoint type, publication year, and MM treatments including thalidomide (Thal), bortezomib (Bort), or lenalidomide (Len). All other covariables used in this multivariate censored-normal regression model have non-significant coefficients, suggesting a weak explanatory power on median OS in the presence of the highly significant TDE regressor (P < 0.0001). These results also suggest a borderline significant positive association (P = 0.06) between trials including patients with relapsed, refractory, or advanced MM, compared with trials including newly diagnosed MM patients. This weak evidence suggests that other factors not included in the regression model may complement the TDE explanatory power in relapsed, refractory, or advanced MM OS. The type of surrogate endpoint and treatment did not impact the explanatory power to median OS, which suggests surrogacy of TTP, PFS, and EFS to OS.

**Table 2:** Effect of median time-dependent endpoints (TDEs) on median overall survival (OS) estimated from a multivariate censored normal regression, by type of TDE

**Covariables**	***β***	**SE**	***P***	**95% CI**
Median TDE, months	2.45	0.38	< 0.0001	1.71 ; 3.20
Females,%	0.05	0.24	0.83	− 0.42 ; 0.52
Median age, years	0.59	0.38	0.12	− 0.16 ; 1.34
Year of publication	0.24	0.32	0.46	− 0.39 ; 0.87
Relapsed, refractory, or advanced MM	14.24	7.60	0.06	− 0.66 ; 29.14
Mixed or not reported type MM	6.95	5.02	0.17	− 2.89 ; 16.78
TDE (time to progression)	5.95	4.16	0.15	− 2.21 ; 14.11
TDE (event-free survival)	− 3.51	4.03	0.38	− 11.42 ; 4.40
Treatment including Thal, Bort, or Len	− 5.98	6.08	0.33	− 17.89 ; 5.94

Note: Includes 163 uncensored observations and 67 right-censored observations. The constant of the model was suppressed due to non-significance.

Abbreviations: *β* Regression coefficient, *Bort* Bortezomib, *CI* Confidence interval *Len* Lenalidomide, *MM*, Multiple myeloma, *SE*, Standard error, *Thal* Thalidomide.

We also tested our modeling technique against a set of alternative specifications and data samples (censored vs uncensored) to assess the robustness of the quantitative relationship between median TDEs and median OS. The departure model identified in Table 3 as base model includes covariables to control for differences in age, gender, and year of publication across studies. Two sets of data were used: sample without censored observations including only those studies reaching median OS (n = 163); and a larger dataset with 67 additional observations including studies not reaching median OS at the last published follow-up (sample with censored observations, n = 230). In this table, all *β* were highly significant (P < 0.0001). The inclusion of TTP and EFS as covariables relative to PFS revealed no statistical differences in the type of TDE but a numeric increment of the estimate coefficient of the linear regression relative to the base model. These results suggest that in trials using PFS, the quantitative relationship between PFS and OS is not statistically different to the comparison between TTP or EFS, and OS. However, caution is recommended in interpreting this result because the numeric differences may point to relevant differences in bigger samples. Augmenting the model with information on MM treatment with Thal, Bort, or Len (sample with censored observations) increased the TDE coefficient from 2.36 to 2.45. Despite these therapies not being statistically significant relative to other treatments, they were retained in the model to control for unobserved differences in trials using newer treatment options compared with older treatments. This does not imply that Thal, Bort, and Len have no effect on OS, but instead suggests that these compounds do not add more explanatory power on median OS other than their effect through median TDE.

**Table 3:** Effect of median time-dependent endpoints (TDEs) on median overall survival estimate based on alternative modeling specifications

**Specification**	**With censored observations (n = 230)**			**Without censored observations (n = 163)**		
	***β***	**SE**	**95% CI**	***β***	**SE**	**95% CI**
Base model (BM)^a^	1.82	0.134	1.56 ; 2.08	2.09	0.206	1.69 ; 2.50
BM without two perturbing observations	1.84	0.137	1.58 ; 2.11	2.16	0.218	1.73 ; 2.58
BM with adjustment for type of patient and type of TDE^b^	2.36	0.344	1.69 ; 3.04	2.64	0.568	1.52 ; 3.75
BM with adjustment for type of patient^c^, type of TDE and treatment including Bort, or Len, or Thal	2.45	0.381	1.71 ; 3.20	2.62	0.558	1.52 ; 3.71

^a^The BM is adjusted for age, gender, and year of publication.

^b^Type of TDE refers to time to progression, progression-free survival, and event-free survival.

^c^Type of MM patient refers to the following groups: newly diagnosed; relapsed, refractory, or advanced; and mixed or not discriminated in the publication.

Abbreviations: *β* Regression coefficient, *Bort* Bortezomib, *CI* Confidence interval, *Len* Lenalidomide, *MM* Multiple myeloma, *SE* Standard error *Thal* Thalidomide.

Overall, the consistency of the estimated values for the effect of median TDE on median OS is evident. The maximum variation in the effect of median TDE on median OS is 34% (*β* = 2.45; 95% CI, 1.71–3.20) relative to the base model (*β* = 1.82; 95% CI, 1.56–2.08).

Results based on the sample with uncensored observations provide higher effect values, most likely related to the study design. In our sample, phase II and phase III randomized controlled trials (RCTs) represent 38.8% and 46.3%, respectively, of the study arms with censored observations, and 23.9% and 55.2%, respectively, of the study arms without censored observations. The results from the regression models including and excluding the two outliers are quite similar, indicating the small effect of these two observations in the analysis.

Additional details of the statistical tests performed to assess the validity of the modeling procedures can be found in the Additional file 1.

#### Prediction of median OS from the observation of median TDE

The detailed predicted median OS and associated 95% CIs based on the observed median TDE for each study arm included in our analysis are presented in the Additional file 1. In general, lower predicted median OS values in the study arms using TTP were found. TTP is the only TDE that does not include death. There was a higher proportion of study arms using TTP as the primary endpoint in the relapsed, refractory, or advanced MM population (46%) compared with 26% and 25% of the study arms evaluating PFS and EFS, respectively. In addition, 35.5%, 63.7%, and 58.0% of the observed median OS are contained in the 95% CI prediction for the TTP, PFS, and EFS, respectively. In approximately one-third of the TTP arms, median OS is under-observed relative to the 95% CI. In the PFS subset, 26.3% of arms reported median OS below the predicted 95% CI and 10% above it. In the EFS subset, 36% of arms report median OS below the predicted 95% CI and 6% above it.

**Figure 2:**
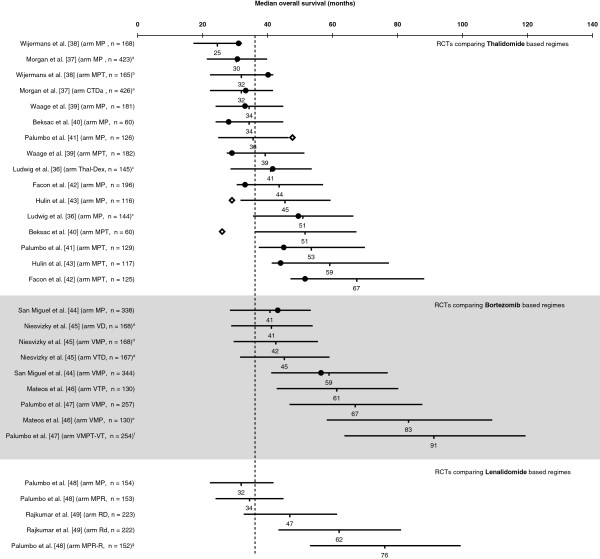
**Predicted median overall survival (OS) versus the observed OS in randomized controlled trials (RCTs) including thalidomide-, bortezomib-, or lenalidomide-based regimens for upfront treatment of multiple myeloma in patients not eligible for transplantation.** Horizontal lines represent estimated 95% confidence interval (CI) for the median OS based on the modeled median time-dependent endpoints from each study arm. Solid circles represent observed median OS contained in the corresponding 95% CI estimate, and lozenges represent observed median OS not contained in the corresponding 95% CI estimate. Where data are from different studies, a direct comparison is not possible as different studies use different patient populations, different study designs, and were conducted at different time points. Vertical dashed line stands for the mean of median OS of the MP arms. ^a^19.5% of trial patients received thalidomide maintenance therapy. ^b^88% of patients received thalidomide maintenance therapy. ^c^18.8% of trial patients received thalidomide maintenance therapy. ^d^33–55% of trial patients received bortezomib maintenance therapy. ^e^35% of trial patients received maintenance therapy with bortezomib plus prednisone and 33.5% with bortezomib plus thalidomide. ^f^66.7% of patients received maintenance therapy with bortezomib plus thalidomide. ^g^100% of patients planned to receive maintenance therapy with lenalidomide. Abbreviations: CTDa, attenuated (low-intensity) cyclophosphamide, thalidomide, dexamethasone; MP, melphalan, prednisone; MPR, melphalan, prednisone, lenalidomide; MPR-R, melphalan, prednisone, lenalidomide followed by maintenance with lenalidomide; MPT, melphalan, prednisone, thalidomide; Rd, lenalidomide, low-dose dexamethasone; RD, lenalidomide, dexamethasone; TD, thalidomide, dexamethasone; VD, bortezomib, dexamethasone; VMP, bortezomib, melphalan, prednisone; VMPT-VT, bortezomib, melphalan, prednisone, thalidomide followed by maintenance with bortezomib, thalidomide; VTP, bortezomib, thalidomide, prednisone.

**Figure 3:**
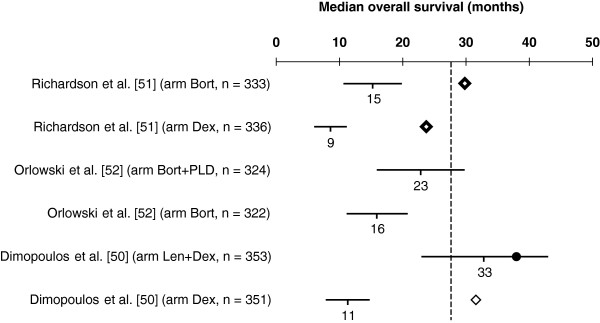
**Predicted median overall survival (OS) versus the observed OS in phase III randomized controlled trials (RCTs) including bortezomib- or lenalidomide-based regimens for the treatment of relapsed/refractory multiple myeloma.** Horizontal lines represent estimated 95% confidence intervals (CIs) for the median OS based on the modeled median time-dependent endpoints from each study arm. Solid circle represents observed median OS contained in the corresponding 95% CI estimate, and lozenges represent the observed median OS not contained in the corresponding 95% CI estimate. Where data are from different studies, a direct comparison is not possible as different studies use different patient populations, different study designs, and were conducted at different time points. Vertical dashed line stands for the mean of median OS of the Dex arms. Abbreviations: Bort, bortezomib; Dex, dexamethasone; Len, lenalidomide; PLD, pegylated liposomal doxorubicin.

**Figure 4:**
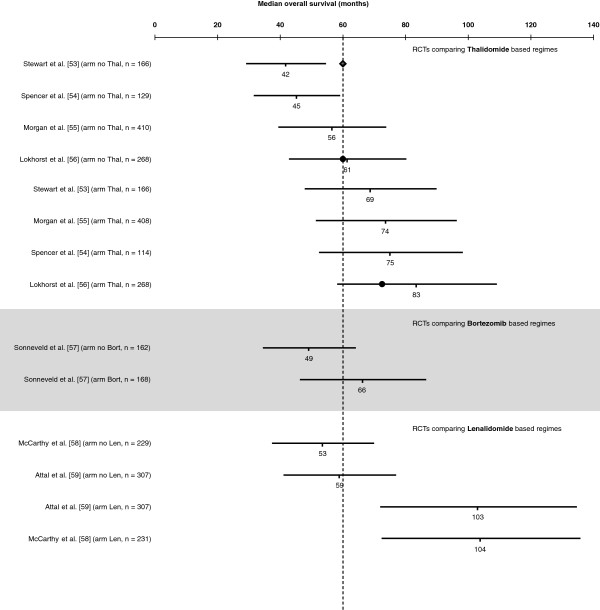
**Predicted median overall survival (OS) versus the observed OS in randomized controlled trials (RCTs) including thalidomide-, bortezomib-, or lenalidomide-based regimens for post-transplantation maintenance therapy in multiple myeloma.** Horizontal lines represent estimated 95% CIs for the median OS based on the modeled median time-dependent endpoints from each study arm. Solid circles represent observed median OS contained in the corresponding 95% CI estimate, and blue lozenge represents observed median OS not contained in the corresponding 95% CI estimate. Vertical dashed line stands for the mean of median OS of the no maintenance arms. Abbreviations: Bort, bortezomib; Dex, dexamethasone; Len, lenalidomide; Thal, thalidomide.

A selection of RCTs retrieved from three recent reviews [33-35] investigating treatment options for newly diagnosed MM in patients not eligible for transplantation [36-49], the relapsed/refractory MM setting [50-52] and post-transplantation maintenance therapy [53-59], respectively, were used to illustrate the practicality of our method in predicting median OS from observed data on median TDE. Figures 2, 3, 4 plot the predicted median OS and associated 95% CIs in comparison with the observed/reported median OS, including those that did not report the median OS due to a short follow-up period. It should be noted that the RCTs presented in these figures are very heterogeneous in their design, patient populations, and treatment options, with some including maintenance therapy. This is not an exhaustive sample of all RCTs, but simply represents a selection of published trials that report median PFS or TTP or EFS.

Since 2007, results from 8 phase III trials investigating Thal combinations in patients with newly diagnosed MM not eligible for transplantation have been reported. With the exception of the studies by Ludwig et al. [36] and Morgan et al. [37], all other studies compare Thal added to melphalan plus prednisone (MPT) with melphalan plus prednisone (MP). The majority of median OS values reported (13/16) are within our 95% CI estimate for OS, confirming the value of TDE in predicting OS (Figure 2). Another practical implication from our method is that once median TTP/PFS/EFS is observed, we can derive an estimate for the median OS even if it has not been reported. The OS estimates can be used to inform decision makers on newer and promising MM treatment regimens, along with other relevant clinical parameters such as acceptable tolerability, favorable safety profile, and sustainable quality-of-life outcomes.

In Figure 3, we present the estimated median OS vs the observed OS in phase III RCTs using Bort- or Len-based regimens for the treatment of relapsed/refractory MM. The only treatment arm reporting median OS within the 95% CI from our model is the Len plus dexamethasone (Dex) arm from the study by Dimopoulos et al. [50]. At first glance, these results suggest that our model is less suitable for predicting median OS in relapsed/refractory MM. However, it should be noted that the effect from subsequent salvage therapies is expected to be more pronounced, with a shorter median TDE, i.e. the faster progression occurs.

A recent review by Reece [35] identified several phase III RCTs of post-transplantation maintenance therapy with Thal, Len, and the proteasome inhibitor Bort. These studies differ substantially in the type of induction regimen, transplantation strategy (single vs tandem) and maintenance scheme (drug combinations, doses, and duration). However, this heterogeneity provided us with the opportunity to further evaluate our model. Figure 4 incorporates updated survival outcomes from these trials and presents estimations for the median OS. Trials identified in Reece [35] that did not report median intermediate TDE were not considered in this figure. The study by Lokhorst et al. [56] was the only one from this set to contribute with data to our regression model. At present, we can estimate an average absolute increase of 27 months, 17 months, and 47 months in median OS for post-transplantation maintenance therapy with Thal, Bort, and Len, respectively.

## Discussion

There is a sound body of evidence suggesting that TDEs such as PFS, TTP, and EFS are appropriate surrogate endpoints for OS in several types of cancer [31,60-71]. However, some conflicting evidence [61,72,73] and some methodological [11], regulatory [74], and conceptual/practical [75] arguments fuel the ongoing discussion about surrogate endpoints in the cancer literature [76-78] and challenge the establishment of TDEs in oncology clinical development [79].

Our study is the first to highlight the value of TDEs in predicting OS in MM and to confirm the recommendations of the American Society of Hematology/US FDA Workshop on Clinical Endpoints in both newly diagnosed or relapsed/refractory MM [17]. We focused our research on estimating the absolute effect of TDEs on OS rather than using a relative measure. We are aware that other assessments of potential surrogate endpoints require a two-step validation process, which involves: 1) establishing that the surrogate endpoint predicts the final endpoint accurately; 2) demonstrating that the effect of treatment on both the surrogate endpoint and the final endpoint is closely correlated [11]. Our methodology, while inherently considering the two last criteria, follows a less formal [80] approach by using regression modeling methods to show that the effect on median OS is captured by the TDE (validation criterion 1) and that adding treatment to the linear predictor does not improve the prediction (validation criterion 2) (i.e. does not improve the fit), hence suggesting that the causal link between treatment and endpoint has been captured by the TDE predictor.

In order to assess our model’s ability to predict OS in different settings, we confronted our estimates with data from studies in relapsed/refractory MM led by Dimopoulos et al. [50] and Richardson et al. [51] (Figure 3). The study by Dimopoulos et al. comparing Len plus Dex with Dex alone reported a hazard ratio (HR) for progression of 0.31 (median 13.4 vs 4.6 months) [81], and for death of 0.71 (median 38.0 vs 31.6 months) [50]. In the study by Richardson et al., which compared Bort to Dex, the reported HR for progression was 0.55 (median 6.2 vs 3.5 months) [82] and for death was 0.77 (median 29.8 vs 23.7 months) [51].

Estimates of median OS using our model suggest an HR for death of 0.34 for Len plus Dex vs Dex alone (median OS 33 vs 11 months), and 0.55 for Bort vs Dex (median OS 15 vs 9 months), assuming event times are exponentially distributed [83]. In this case, the treatment effect on TTP would explain more than 90% of the treatment effect on OS for both Len plus Dex and for Bort.

It has been argued that OS is not a realistic endpoint in this setting [84], especially considering the ever increasing availability of new, effective drugs that can be used as salvage therapies [8,85] which may mask the real survival differences between treatment arms. Statistical methods to correct for bias resulting from non-informative censoring (crossover and subsequent treatment options) in survival analysis are increasingly popular [86,87]. In a recent paper by Ishak et al. [88], information from trials conducted by the Medical Research Council (United Kingdom) was used to calibrate survival regression analyses in order to reproduce survival estimates corrected for patient crossover in clinical trials. These authors present a median OS of 11.6 months (95% CI, 9.5–14.2) for patients with > 1 prior therapy randomized to Dex [50], which is similar to our estimate of 11.3 months. Furthermore, in a survival analysis adjusted for crossover in the APEX trial, Pacou et al. report an OS HR of 0.59 for Bort relative to Dex [89], which is also very similar to the value derived from our model (HR 0.55), suggesting that our model performs accurately in trials with substantial crossover. Nonetheless, caution is recommended for extrapolation outside the context of our sample because more mature data on more recent clinical trials and future research in this topic is clearly needed.

We provide a more straightforward way of calculating the expected effect of treatment on median OS (prior to the observation of mature OS data), by estimating an absolute rather than a relative measure for the quantitative relationship between the median TDE and median OS. This regression model recognizes the influence of subsequent therapies because it estimates a mean effect of median TDE on median OS using OS data published in the literature, which is uncorrected for the effect of non-randomized subsequent treatment options. We estimated an average increment of 2.45 months in median OS for each additional month of median TDE. As previously highlighted, these estimates are valuable to assess the expected impact of treatments on median OS, for example in trials of newly diagnosed MM where median OS may not be reached for several years.

Information on survival is essential for clinical trial design [90], accelerated approvals for new drugs [91], indirect drug comparisons, and economic considerations (e.g. formulary inclusion and other reimbursement decisions), particularly in the absence of head-to-head comparative clinical trials. Such information may help clinicians select the most suitable treatment options for MM patients.

Other studies examining the relationship between TDEs and OS have been reported in metastatic colorectal cancer (mCRC) [31,62] and in metastatic breast cancer [72]. In mCRC, there was a strong association between PFS and OS [31,62], with similar correlation coefficients as obtained in our analysis of MM patients [62]. In metastatic breast cancer, no particular endpoint was determined to be an adequate surrogate for OS [72]. The different conclusions from studies in breast cancer, mCRC, and MM emphasize the fact that appropriate TDEs cannot be generalized in oncology, and their validity depends on tumor type.

The following caveats should be considered when interpreting our results. Although it seems reasonable to question the endogeneity of TDEs as an explanatory variable for OS, this issue has not been addressed in the MM literature. The methodology presented here attempts to solve the endogeneity problem, but its applicability depends on the availability of valid instruments.

In this analysis, TDEs include three distinct surrogate endpoints; TTP, PFS, and EFS. The estimated relationship between the TDE and OS represents the relationship between an “average” TDE and OS. Although no statistical differences have been found in modeled OS by the type of TDE, the value of the information is limited. Further studies are necessary, particularly to clarify the data from studies using TTP, both because of the competing risk estimation problems [92] and the arguments against the use of TTP [7]. Testing could be performed by either modeling each of the subsamples or by including an interaction term between the TDE and type of surrogate endpoint marker in the regression. In the current analysis, no testing could be performed due to the sample size and need for additional (valid) instruments.

Our analysis includes therapies available over a period of 40 years that demonstrated a wide range of efficacy levels. We attempted to control these differences by using publication year as a covariate. In addition, our censored analysis omitted treatment arms with proportionally longer median OS and therefore may not reflect adequately the impact of newer, more effective therapies. Finally, the majority of the studies did not report whether data for OS included patients who were allowed to crossover between treatment arms. Study designs that include automatic treatment crossover can obscure differences in OS, due to the benefit achieved from subsequent treatments [8].

## Conclusion

In conclusion, our analysis confirms the potential value of TDEs (TTP, PFS, and EFS) in predicting OS in patients with MM. Additional research is welcomed to refine this model or to identify alternative complementary statistical models. Until such models are available and validated, the quantitative relationship presented here may be of value in the design of clinical trials, indirect drug comparisons, and economic assessment of new MM drugs.

## Competing interests

The authors declare they have no competing interests.

## Authors’ contributions

JF coordinated the research project, participated in the conception and design of the study, participated in results interpretation and discussion, and drafted the manuscript. FA participated in design of the study, performed the statistical analysis, and participated in results interpretation and discussion. JMA participated in design of the study, coordinated the literature review, and participated in results interpretation and discussion. FJMC participated in design of the study and coordinated the literature review. DF participated in the literature review and in data extraction. ABSP participated in the conception and design of the study and participated in results interpretation and discussion. RR participated in the literature review and in data extraction. JFRR participated in the literature review and in data extraction. All authors read and approved the final manuscript.

## Pre-publication history

The pre-publication history for this paper can be accessed here:

http://www.biomedcentral.com/1471-2407/13/122/prepub

## Supplementary Material

Additional file 1 Additional Results. Click here for file
