# Supplementary material for: Time-dependent endpoints as predictors of overall survival in multiple myeloma
Source: BMC Cancer. 2013 Mar 16;13:122. doi: 10.1186/1471-2407-13-122 (PMC3607860; doi:10.1186/1471-2407-13-122)
Supplement: Additional file 1 — Additional Results. [file 1471-2407-13-122-S1.docx]

**Additional file 1 (Section 1)**

**Additional Results**

**Modeling the effect of TDE on OS**

Normality of the dependent variable (median OS), was tested using an overall statistic test, which combines a test for normality based on skewness and another based on kurtosis, and it could not be rejected [1]. Two mild and no severe outliers were detected using the Grubbs test [2]. In order to evaluate the impact of these outliers, the model was re-run without the identified two outliers. The estimates for the relation between median OS and TDS increased only slightly (1.1%). As such, outliers were included in the analysis.

Based on the Durbin-Wu-Hausman test for the endogeneity of the TDS regressor, the null hypothesis of zero correlation was rejected, suggesting the presence of endogeneity and the feasibility of instrumental variables approach.

Both the White/Koenker nR2 [3] and the Breusch-Pagan/Godfrey/Cook-Weisberg tests for IV heteroskedasticity rejected the null of constant variance, pointing to the adequacy of the GMM estimator, as discussed in the methods section.

Linearity of the functional form was tested using the heteroskedastic-robust Ramsey/Pesaran-Taylor RESET test. The null hypothesis of no omitted variables, or more specifically of the linearity of the functional form, could not be rejected. When the Variance Inflation Factor (VIF) was used to test for multicollinearity, no value above 5 was obtained for any of the independent variables thereby suggesting its absence. The mean value of the VIF statistics was 2.49.

In light of this evidence, the final model has the median OS as the dependent variable and a constant and median TDE as explanatory variables. In that model, TDE is instrumentalized by: OS at 12 months, the proportion of females, median age, year of publication and dummies characterizing patients with regard to previous MM treatment, type of TDE, and treatment with new multiple myeloma drugs (Bortezomib, Lenalidomide, and Thalidomide).

A set of statistical tests were performed with the objective of assessing the quality of the instruments used in the two steps regression. Regarding the instruments’ explanatory power, several tests are available and results were consistent in rejecting the null of weak correlation between the instruments and the endogenous regressor (*Shea Partial R^2^* = *0.4759; F(9,153) = 26.97;* P *< 0.0001)*; Anderson canononical correlation: (*χ^2^ (9) = 105.3;* P *< 0.001)*, Cragg-Donald N*CDEV: (χ^2^ *(9) = 147.99;* P *< 0.001)*; Robust chi-square statistic: (*χ^2^(9) = 258.59;* P *<0.001)*. Regarding the orthogonality of the 12-months OS instrument, the null of exogeneity could not be rejected (Hanssen J statistic for over identification test of all instruments): *χ^2^(8) = 13.428;* P *= 0.0979*; *Hanssen J statistic* excluding suspect orthogonality conditions: *χ^2^(7) = 0.2131;* P *= 0.2131*; C-statistic: *χ^2^(1) = 3.839;* P *= 0.0501)*. The total uncentered *R^2^* of the first step regression was 0.8424, and the Pseudo *R^2^* = 0.2382 of the second step censored regression was 0.2382 (AIC=1166.947; BIC=1197.89).

**References**

1. D'Agostino RB, Belanger AJ, D'Agostino RB. Jr.: **A suggestion for using powerful and informative tests of normality**. *The American Statistician* 1990, **44**:316-321.
2. NIST/SEMATECH *e-Handbook of Statistical Methods* 2012: <http://www.itl.nist.gov/div898/handbook>
3. Koenker R.: **A note on Studentizing a test for heteroskedasticity**. *Journal of Econometrics* 1981, **17**:107-112.

**Additional file 1 (Section 2)**

Studies excluded after evaluation of full text eg, reviews of the literature, did not report median TDE or adequate survival data (n = 359)

Potentially relevant articles identified and screened for retrieval (n = 1.061)

Citations excluded (n = 550), eg, letters to editor, comments, retrospective studies

Potentially eligible articles (n = 511)

Relevant studies for the analysis (n = 152)

230 study arms

- RCT phase III (n = 121*)
- Phase II (n = 65)
- Cohort study (n = 25)
- Quasi experimental (n = 11)
- Case series (n = 8)

**Figure 1. Flow of Included and Excluded Studies**. One investigator assessed titles and abstracts to determine if each article met the predetermined eligibility criteria. If the title or abstract were ambiguous, the full text of the article was reviewed before the study was excluded; full text reading of all studies was performed by four independent reviewers. Any article that did not meet all inclusion criteria or met any of the predetermined exclusion criteria was excluded. Whenever doubts arose regarding the eligibility of a particular study, the paper was independently assessed by a second reviewer, and any disagreements and their resolution recorded. When consensus could not be reached between these two reviewers, the study was assessed by a third investigator, whose decision was considered final; all excluded studies and the reasons for exclusion were recorded.

Figure 2. Predicted Median Overall Survival (OS) and Associated 95% Confidence Intervals (CIs) Versus the Observed OS. Solid horizontal lines represent estimated 95% CI for the median OS based on the modeled median time-dependent surrogates from each study arm. Red solid circles represent observed median OS contained in the corresponding 95% CI estimate, and blue circles represent observed median OS not contained in the corresponding 95% CI estimate. Each study arm treatment and number of patients is identified in conjunction with the first author and year of publication. This figure is presented without the two outliers on progression-free survival surrogate and without the predicted 95% CI for trial arms with censored median OS.

**Table 1.** PubMed Search Filter for the Systematic Literature Review

| 1 | myeloma |
| --- | --- |
| 2 | myelom* |
| 3 | "Multiple Myeloma" [MeSh] |
| 4 | survival |
| 5 | surviv* |
| 6 | death |
| 7 | "Survival" [MeSh] |
| 8 | "Mortality" [MeSh] |
| 9 | progression |
| 10 | progress* |
| 11 | "Disease Progression" [MeSh] |
| 12 | event-free |
| 13 | event-free* |
| 14 | randomized controlled trial [pt] OR controlled clinical trial [pt] OR randomized controlled trials [mh] OR random allocation [mh] OR double-blind method [mh] OR single-blind method [mh] OR clinical trial [pt] OR clinical trials [mh] OR ("clinical trial" [tw]) OR ((singl* [tw] OR doubl* [tw] OR trebl* [tw] OR tripl* [tw]) AND (mask* [tw] OR blind* [tw])) OR (placebos [mh] OR placebo* [tw] OR random* [tw] OR research design [mh:noexp] OR comparative study [pt] OR evaluation studies [pt] OR follow-up studies [mh] OR prospective studies [mh] OR control* [tw] OR prospectiv* [tw] OR volunteer* [tw]) NOT (animals [mh] NOT humans [mh]) |
| 15 | 1 or 2 or 3 |
| 16 | 4 or 5 or 6 or 7 or 8 |
| 17 | 9 or 10 or 11 or 12 or 13 |
| 18 | 15 and 16 and 17 and 14 |

**Table 2.** List of Studies Included in the Analysis

| **Reference** | **Period of Observation** | **Participants per  Study Arm** | **Treatment per Study Arm** | **Type of TDE** |
| --- | --- | --- | --- | --- |
| Abdelkefi et al., 2008 | 2003-2006 | 97 | MEL+THA | PFS |
| Abraham et al., 1999 | 1992-1998 | 100 | MEL160+TBI | EFS |
| Anderson et al., 1993 | 1987-1992 | 26 | MEL+CYC+TBI | PFS |
| Arora et al., 2004 | 1993-2002 | 72 | GCSF or GMCSF | PFS |
| Attal et al., 1996 | 1990-1993 | 100 // 100 | VMCP+BVAP // MEL140+TBI | EFS |
| Attal et al., 2003 | 1994-1997 | 199 // 200 | MEL140+TBI //MEL140 | EFS |
| Attal et al., 2006 | 2000-2003 | 200 // 196 // 201 | None // PAM // PAM+THA | EFS |
| Badros et al., 2001a | 1996-2000 | 31 | MEL | EFS |
|  |  |  |  | EFS |
| Badros et al., 2001b | 1992-1999 | 39 // 31 | MEL // MEL | EFS |
| Badros et al., 2005 | 2002-2004 | 33 | G3139+DEX+THA | PFS |
| Ballestrero et al., 2002 | NR | 20 | CEM+MEL | EFS |
| Bang et al., 2003 | 1997-2002 | 80 | MEL200 | EFS |
| Barbui et al., 2002 | 1997-2000 | 29 // 31 | MEL // MEL | EFS |
| Barlogie et al., 1998 | 1995-1997 | 24 // 8 // 18 // 37 | MEL100+GMCSF // MEL140 // | EFS |
|  |  |  | THIO+TBI // MEL140+TBI |  |
| Barlogie et al., 2006a | NR | 261 // 255 | MEL140+TBI // VBMCP | PFS |
| Barlogie et al., 2006b | 1998-2004 | 345 // 323 | MEL // MEL+THA | EFS |
| Barlogie et al., 2006c | 1990-1995 | 231 | TT1 | EFS |
| Baz et al., 2006 | NR | 62 | DVd+LEN | PFS |
| Beksac et al., 2010 | 2006-NR | 58 // 57 | MPT // MP | EFS |
| Belch et al., 1988 | 1977-1984 | 93 // 92 | MP // None | TTP |
| Berenson et al., 2002 | 1993-1997 | 65 // 61 | P10 // P50 | PFS |
| Berenson et al., 2006 | 2005 | 65 | MAC | PFS |
| Berenson et al., 2007 | NR | 22 | ABC | PFS |
| Berz et al., 2009 | NR | 13 | MEL | TTP |
| Bladé et al., 2000 | 1990-1998 | 31 // 33 | HDT // SC | EFS |
| Bladé et al., 2005 | 1994-1999 | 83 // 81 | VBMCP/VBAD // HDT | PFS |
| Bourhis et al., 2007 | 1995-1999 | 56 // 55 | MEL+TBI // MEL+TBI | EFS |
| Bremer, 2002 | 1993-1998 | 25 | BE | PFS |
| Browman et al., 1995 | 1987-1992 | 85 // 91 | MP+IFN // MP | PFS |
| Chen et al., 2006 | 1998-2000 | 28 | DEX+MEL+P+CYC+IFN | EFS |
| Child et al., 2003 | 1993-2000 | 200 // 201 | DOX+CAR+CYC+MEL+PR // DOX+VIN+MEP+CYC+G-CSF+MEL | PFS |
| Ciolli et al., 2008 | 2004-NR | 28 // 42 | VTD // VTD-PLD | TTP |
| Clark et al., 2002 | 1995-1999 | 26 | DHAP+CYC+MB | EFS |
| Comenzo et al., 2006 | 1999-2003 | 49 | HDM+CAR | PFS |
| Cook et al., 2004 | 1996-2002 | 54 // 52 | VAD // Z-DEX | PFS |
| Corso et al., 2007 | 2000-2004 | 65 // 122 | MEL200 // MEL200 | EFS |
| Davies et al., 2001 | 1993-1998 | 96 | MEL | PFS |
| Desikan et al., 2000 | NR | 19 // 24 // 43 | MEL+CYC // MEL+TBI // MEL200 | EFS |
| Dimopoulos et al., 2001 | 1999-2000 | 44 | THA+DEX | TTP |
| Dimopoulos et al., 2004 | 2000-2002 | 53 | CTD | TTP |
| Dimopoulos et al., 2007 | 2003-2004 | 176 // 175 | LEN+DEX // DEX | TTP |
| Dingli et al., 2005 | NR | 21 | THA+DEX | TTP |
| Dispenzieri et al., 2010 | 2004-2005 | 42 | BOR | PFS |
| Drayson et al., 1998 | 1989-1995 | 143 // 141 | INF // None | PFS |
| Dumontet et al., 2003 | 1997-1998 | 21 | FOT | TTP |
| Einsele et al., 2003 | 1994-1998 | 89 | BUC+TBI | EFS |
| Eom et al., 2006 | 1996-2005 | 17 | MEL | PFS |
| Facon et al., 2006 | 1995-1998 | 122 // 118 // 127 // 121 | MP // M+DEX // DEX // DEX-IFN | PFS |
| Fassas et al., 2002 | 1996-1999 | 75 | DECP | EFS |
| Fenk et al., 2005 | 1997-1999 | 30 // 26 | MEL200 // HD-IMC | TTP |
| Fermand et al., 1993 | 1986-1991 | 63 | CAR+MEL+ETP+TBI | EFS |
| Fermand et al., 1998 | 1990-1997 | 91 | HDT | EFS |
| Fermand et al., 2005 | 1991-1998 | 94 // 96 | HDT // VMCP | EFS |
| Fosså et al., 1998 | 1991-1996 | 25 | VECD | PFS |
| Friedenberg et al., 2006 | 1997-2000 | 48 // 46 | VAD // VAD+DPV | PFS |
| Garcia-Sanz et al., 2006 | 2001-2003 | 30 | MEL+PLD+P | PFS |
| Ghosh et al., 2011 | 2004-2006 | 27 | BT | PFS |
| Gianni et al., 1994 | 1989-1991 | 13 | MEL+TBI | PFS |
| Giralt et al., 2003 | 1998-2000 | 88 | MEL | EFS |
| Gojo et al., 2006 | 1997-2001 | 103 | CAR+MEL+GEM | EFS |
| Harousseau et al., 1992 | 1984-1990 | 53 // 44 | MEL140 // MEL140 | PFS |
| Harousseau et al., 2010 | 2005-2009 | 242 // 240 | VAD // BOR+DEX | PFS |
| Hernández et al., 2004 | NR | 87 // 83 | MP // M+DEX | EFS |
| Hjorth et al., 1993 | 1983-1988 | 25 // 25 | MP // MP | PFS |
| Hjorth et al., 1996 | 1990-1994 | 297 // 286 | MP // MP+INF | EFS |
| Horvath et al., 2004 | 1995-1997 | 34 | CYE+MB | EFS |
| Huijgens et al., 2001 | 1992-1997 | 90 // 49 | MEL140 // BUS+CYC | EFS |
| Hulin et al., 2009 | 2002-2006 | 117 // 115 | MP // MPT | PFS |
| Hussein et al., 2002 | NR | 33 | DVd | TTP |
| Kars et al., 1997 | 1985-1994 | 33 // 31 | VAD+INF // VAD | PFS |
| Keldsen et al., 1993 | 1987-1989 | 77 // 74 | MVP // MP | TTP |
| Kneppers et al., 2010 | 2007-2007 | 117 | LEN | PFS e TTP |
| Kropff et al., 2003 | 2000-2001 | 60 | HyperCDT | EFS |
| Kropff et al., 2007 | 2004-2005 | 54 | BOR+DEX+CY | EFS |
| Kumar et al., 2003a | 1999-2000 | 32 | THA | PFS |
| Kumar et al., 2003b | 1990-2002 | 50 | HDT | PFS |
| Kyle et al., 2009 | 1994-2002 | 111 // 112 | VBMCP // VBMCP+IFN | PFS |
| Kyriakou et al., 2005 | 2002-2004 | 52 | CTD | EFS |
| Lacy et al., 2007 | 2004-2004 | 21 | LEN+DEX | TTP |
| Lahuerta et al., 2000 | 1990-1999 | 245 // 102 // 93 | MEL200 // MEL+TBI // MEL+BUS | EFS |
| Lahuerta et al., 2003 | 1994-1999 | 88 | MEL+CBV | EFS |
| Lee et al., 2003 | 1998-2002 | 33 | MEL200 | EFS |
| Lee et al., 2010 | 2005-2007 | 40 | PAD-TD | PFS |
| Lenhoff et al., 2006 | 1994-1997 | 313 | MEL | EFS |
| Lokhorst et al., 2010 | 2001-2005 | 268 // 268 | VAD // TAD | EFS |
| Ludwig et al., 1995 | NR | 125 // 131 | VMCP+IFN // VMCP | TTP |
| Ludwig et al., 2005 | 1994-2001 | 148 // 144 | VMC-Pcont // VMCP | PFS |
| Ludwig et al., 2009 | 2001-2007 | 145 // 143 | THA+DEX // M+PR | PFS |
| Ludwig et al., 2010 | 2001-2007 | 145 // 144 | IFN // TI | PFS |
| Martino et al., 2007 | NR | 17 | MEL+THA | PFS |
| Mellqvist et al., 2008 | 2001-2003 | 156 // 158 | VAD // CYC+DEX | EFS |
| Mileshkin et al., 2003 | 1999 | 75 | THA | PFS |
| Moreau et al., 2002 | 1995-1999 | 140 // 142 | MEL140+TBI // MEL200 | EFS |
| Moreau et al., 2006 | 2000-2004 | 85 // 81 | M+DEX // M+DEX+BE8 | EFS |
| Offidani et al., 1998 | 1989-1994 | 27 // 25 | INF // INF | PFS |
| Offidani et al., 2002 | 1999-2000 | 10 | GEM+CIS | TTP |
| Offidani et al., 2004 | 2000-2002 | 23 | THA | PFS |
| Offidani et al., 2006 | 2003-2005 | 50 | DEX+ PLD+THA | PFS |
| Offidani et al., 2009 | NR | 51 // 52 | DEX-IFN // THA+DEX | PFS |
| Orlowski et al., 2007 | 2004-2006 | 322 // 324 | BOR // BOR+PLD | TTP |
| Ossenkoppele et al., 1996 | 1992-1994 | 30 | MEL140+G-CSF | TTP |
| Palumbo et al., 1993 | 1988-1991 | 28 | INF+GLU | PFS |
| Palumbo et al., 1999 | 1990-1997 | 71 | MEL100 | EFS |
| Palumbo et al., 2004a | 1999-2001 | 58 | THA+DEX | PFS |
| Palumbo et al., 2004b | 1997-2000 | 95 // 99 | MEL100 // MP | EFS |
| Palumbo et al., 2005 | NR | 49 | MPT | EFS |
| Palumbo et al., 2006a | 2002-2005 | 24 | MPT | TTP |
| Palumbo et al., 2006b | 2002-2005 | 126 | MP | EFS |
| Palumbo et al., 2007 | 2004-2005 | 30 | VMPT | PFS |
| Palumbo et al., 2008 | 2005-2006 | 64 | PAD | EFS |
| Palumbo et al., 2010 | 2007-2008 | 44 | LMPT | PFS |
| Peest et al., 1995 | 1988-1991 | 52 // 65 | INF // None | PFS |
| Pönisch et al., 2006 | 1994-1999 | 68 // 63 | BP // MP | TTP |
| Powles et al., 2000 | 1995-1998 | 17 | ALLIC | EFS |
| Prince et al., 2005 | 2001-2004 | 66 | THA+CELECOXIB | PFS |
| Putkonen et al., 2005 | 1992-2003 | 73 | MEL | PFS |
| Rajkumar et al., 2008 | 2003-2005 | 234 // 232 | THA+DEX // DEX | TTP |
| Rajkumar et al., 2010 | 2004-2006 | 223 // 222 | LEN+DEX (high dose // LEN+DEX (low-dose) – comentário!! | TTP e PFS |
| Remes et al., 2003 | 1993-1998 | 39 // 25 | MEL // MEL | PFS |
| Reynolds et al., 2001 | 1994-1999 | 35 | BUS+CYC | PFS |
| Ria et al., 2004 | NR | 16 // 14 | MEL // MEL+BUS | PFS |
| Richardson et al., 2003 | 2001 | 193 | BOR | TTP |
| Richardson et al., 2004 | 1999-2001 | 26 | THA | PFS |
| Richardson et al., 2006a | NR | 202 | BOR+DEX | TTP |
| Richardson et al., 2006b | 2002-2003 | 102 | LEN+DEX | TTP |
| Richardson et al., 2007 | 2002-2003 | 333 // 336 | BOR // DEX | TTP |
| Richardson et al., 2009 | NR | 222 | LEN | TTP e PFS |
| Rifkin et al., 2006 | 2001-2003 | 97 // 95 | DVd // VAD | TTP |
| Sahebi et al., 2006 | 2001-2004 | 29 | MEL+THA | PFS |
| Salmon et al., 1994 | 1985-1990 | 97 // 96 | INF // None | EFS |
| Salmon et al., 1998 | 1990-1993 | 45 // 44 | INF // INF+P | PFS |
| San Miguel et al., 2008 | 2004-2006 | 344 // 338 | BOR+MP // MP | TTP |
| Schaar et al., 2005 | 1991-1997 | 46 // 44 | INF // MP | PFS |
| Schey et al., 2003 | NR | 69 | THA | PFS |
| Segeren et al., 2003 | 1995-2000 | 129 // 132 | MEL140 // MEL | TTP |
| Sharma et al., 2007 | 2000-2004 | 34 | M+DEX | PFS |
| Shustik et al., 2006 | 1995-2003 | 234 // 232 | MP // M+DEX | PFS |
| Singhal et al., 1999 | 1997-1998 | 84 | THA | EFS |
| Sonneveld et al., 2001 | NR | 41 // 34 | VAD // VAD+CYA | PFS |
| Sonneveld et al., 2007 | 1995-2000 | 148 // 155 | MEL140 // MEL+CYC+TBI | PFS |
| Spencer et al., 2004 | 1997-2000 | 35 | MEL200 | PFS |
| Stewart et al., 2001 | 1995-1996 | 93 // 97 | BUS+CYC // BUS+CYC | PFS |
| Stewart et al., 2004 | NR | 67 | PT | PFS |
| Suvannasankha et al., 2006 | 2003-2005 | 29 | BOR+MP | TTP |
| Takenaka et al., 2004 | 1993-1998 | 107 // 103 | MCNU+COP+MP // COP+MP | PFS |
| Vesole et al., 1999 | 1991-1996 | 66 | MEL100 | PFS |
| Waage et al., 2010 | 2002-2007 | 182 // 175 | MPT // MP | PFS |
| Weber et al., 2007 | 2003-2006 | 177 // 176 | LEN+DEX // DEX | TTP |
| Wijermans et al., 2010 | 2002-2007 | 168 // 165 | MP // MPT | EFS |
| Yakoub-Agha et al., 2002 | 1999-2000 | 83 | THA | EFS |
| Zervas et al., 2007 | 2002-2006 | 115 | VAD-doxil | PFS |
| Zonder et al., 2010 | 2004-2007 | 97 // 95 | LEN+DEX // DEX | PFS |

BOR, bortezomib; BUS, busulfan; CAR, carmustine; CIS, cisplatin; CTD, cyclosphosphamide + thalidomide + dexamethasone; CYC, cyclophosphamide; DEX, dexamethasone; DHAP, dexamethasone + high-dose cytarabine + cisplatin; DOX, doxorubicin; EFS, event-free survival; ETP, etoposide; G-CSF, granulocyte colony stimulating factor; GEM, gemcitabine; GM-CSF, granulocyte macrophage-colony stimulating factor; IFN, interferon; LEN, lenalidomide; MEL, melphalan; MP, melphalan + prednisone; MPT, melphalan + prednisone +

thalidomide; NR, not reported; PFS, progression-free survival; TBI, total body irradiation; THA, thalidomide; TTP, time to progression; VAD, vincristine + adriamycin + dexamethasone; VBAD, vincristine, carmustine, doxorubicin, dexamethasone; VBMCP, vincristine + carmustine + melphalan, cyclophosphamide + prednisone; VECD; vincristine + epirubicin + cyclophosphamide + dexamethasone; VIN, vincristine; VMCP; vincristine + melphalan + cyclophosphamide + prednisolone; Z-DEX, idarubicin + dexamethasone.

**References for Studies Used in the Estimation Model**

1. Abdelkefi A, Ladeb S, Torjman L, et al: Single autologous stem cell transplantation followed by maintenance therapy with thalidomide is superior to double autologous transplantation in multiple myeloma: results of a multicenter randomized clinical trial. Blood 111:1805-1810, 2008
2. Abraham R, Chen C, Tsang R, et al: Intensification of the stem cell transplant induction regimen results in increased treatment-related mortality without improved outcome in multiple myeloma. Bone Marrow Transplant 24:1291-1297, 1999
3. Anderson KC, Andersen J, Soiffer R, et al: Monoclonal antibody-purged bone marrow transplantation therapy for multiple myeloma. Blood 82:2568-2576, 1993
4. Arora M, Burns LJ, Barker JN, et al: Randomized comparison of granulocyte colony-stimulating factor versus granulocyte-macrophage colony-stimulating factor plus intensive chemotherapy for peripheral blood stem cell mobilization and autologous transplantation in multiple myeloma. Biol Blood Marrow Transplant 10:395-404, 2004
5. Attal M, Harousseau JL, Stoppa AM, et al: A prospective, randomized trial of autologous bone marrow transplantation and chemotherapy in multiple myeloma. Intergroupe Français du Myélome. N Engl J Med 335:91-97, 1996
6. Attal M, Harousseau JL, Facon T, et al: Single versus double autologous stem-cell transplantation for multiple myeloma. N Engl J Med 349:2495-2502, 2003
7. Attal M, Harousseau JL, Leyvraz S, et al: Maintenance therapy with thalidomide improves survival in patients with multiple myeloma. Blood 108:3289-3294, 2006
8. Badros A, Barlogie B, Siegel E, et al: Results of autologous stem cell transplant in multiple myeloma patients with renal failure. Br J Haematol 114:822-829, 2001a
9. Badros A, Barlogie B, Siegel E, et al: Autologous stem cell transplantation in elderly multiple myeloma patients over the age of 70 years. Br J Haematol 114:600-607, 2001b
10. Badros AZ, Goloubeva O, Rapoport AP, et al: Phase II study of G3139, a Bcl-2 antisense oligonucleotide, in combination with dexamethasone and thalidomide in relapsed multiple myeloma patients. J Clin Oncol 23:4089-4099, 2005
11. Ballestrero A, Ferrando F, Miglino M, et al: Three-step high-dose sequential chemotherapy in patients with newly diagnosed multiple myeloma. Eur J Haematol 68:101-106, 2002
12. Bang SM, Cho EK, Suh C, et al: High dose therapy followed by autologous peripheral blood stem cell transplantation as a first line treatment for multiple myeloma: a Korean Multicenter Study. J Korean Med Sci 18:673-678, 2003
13. Barbui AM, Galli M, Dotti G, et al: Negative selection of peripheral blood stem cells to support a tandem autologous transplantation programme in multiple myeloma. Br J Haematol 116:202-210, 2002
14. Barlogie B, Jagannath S, Naucke S, et al: Long-term follow-up after high-dose therapy for high-risk multiple myeloma. Bone Marrow Transplant 21:1101-1107, 1998
15. Barlogie B, Kyle RA, Anderson KC, et al: Standard chemotherapy compared with high-dose chemoradiotherapy for multiple myeloma: final results of phase III US Intergroup Trial S9321. J Clin Oncol 24:929-936, 2006a
16. Barlogie B, Tricot G, Anaissie E, et al: Thalidomide and hematopoietic-cell transplantation for multiple myeloma. N Engl J Med 354:1021-1030, 2006b
17. Barlogie B, Tricot GJ, van Rhee F, et al: Long-term outcome results of the first tandem autotransplant trial for multiple myeloma. Br J Haematol 135:158-164, 2006c
18. Baz R, Walker E, Karam MA, et al: Lenalidomide and pegylated liposomal doxorubicin-based chemotherapy for relapsed or refractory multiple myeloma: safety and efficacy. Ann Oncol 17:1766-1771, 2006
19. Beksac, M., R. Haznedar, T. Firatli-Tuglular, et al., Addition of thalidomide to oral melphalan/prednisone in patients with multiple myeloma not eligible for transplantation: results of a randomized trial from the Turkish Myeloma Study Group. Eur J Haematol, 2010. 86(1): p. 16-22.
20. Belch A, Shelley W, Bergsagel D, et al: A randomized trial of maintenance versus no maintenance melphalan and prednisone in responding multiple myeloma patients. Br J Cancer 57:94-99, 1988
21. Berenson JR, Crowley JJ, Grogan TM, et al: Maintenance therapy with alternate-day prednisone improves survival in multiple myeloma patients. Blood 99:3163-3168, 2002
22. Berenson JR, Boccia R, Siegel D, et al: Efficacy and safety of melphalan, arsenic trioxide and ascorbic acid combination therapy in patients with relapsed or refractory multiple myeloma: a prospective, multicentre, phase II, single-arm study. Br J Haematol 135:174-183, 2006
23. Berenson JR, Matous J, Swift RA, et al: A phase I/II study of arsenic trioxide/bortezomib/ascorbic acid combination therapy for the treatment of relapsed or refractory multiple myeloma. Clin Cancer Res 13:1762-1768, 2007
24. Berz, D., G.A. Colvin, E.M. McCormack, et al., Triple MEL100 therapy in multiple myeloma. Transplant Proc, 2009. 41(9): p. 3863-7.
25. Bladé J, Esteve J, Rives S, et al: High-dose therapy autotransplantation/intensification vs continued standard chemotherapy in multiple myeloma in first remission. Results of a non-randomized study from a single institution. Bone Marrow Transplant 26:845-849, 2000
26. Bladé J, Rosiñol L, Sureda A, et al: High-dose therapy intensification compared with continued standard chemotherapy in multiple myeloma patients responding to the initial chemotherapy: long-term results from a prospective randomized trial from the Spanish cooperative group PETHEMA. Blood 106:3755-3759, 2005
27. Bourhis JH, Bouko Y, Koscielny S, et al: Relapse risk after autologous transplantation in patients with newly diagnosed myeloma is not related with infused tumor cell load and the outcome is not improved by CD34+ cell selection: long term follow-up of an EBMT phase III randomized study. Haematologica 92:1083-1090, 2007
28. Bremer K: High rates of long-lasting remissions after 5-day bendamustine chemotherapy cycles in pre-treated low-grade non-Hodgkin’s-lymphomas. J Cancer Res Clin Oncol 128:603-609, 2002
29. Browman GP, Bergsagel D, Sicheri D, et al: Randomized trial of interferon maintenance in multiple myeloma: a study of the National Cancer Institute of Canada Clinical Trials Group. J Clin Oncol 13:2354-2360, 1995
30. Chen CI, Nanji S, Prabhu A, et al: Sequential, cycling maintenance therapy for post transplant multiple myeloma. Bone Marrow Transplant 37:89-94, 2006
31. Child JA, Morgan GJ, Davies FE, et al: High-dose chemotherapy with hematopoietic stem-cell rescue for multiple myeloma. N Engl J Med 348:1875-1883, 2003
32. Ciolli, S., F. Leoni, C. Casini, et al., The addition of liposomal doxorubicin to bortezomib, thalidomide and dexamethasone significantly improves clinical outcome of advanced multiple myeloma. Br J Haematol, 2008. 141(6): p. 814-9.
33. Clark AD, Douglas KW, Mitchell LD, et al: Dose escalation therapy in previously untreated patients with multiple myeloma following Z-Dex induction treatment. Br J Haematol 117:605-612, 2002
34. Comenzo RL, Hassoun H, Kewalramani T, et al: Results of a phase I/II trial adding carmustine (300 mg/m2) to melphalan (200 mg/m2) in multiple myeloma patients undergoing autologous stem cell transplantation. Leukemia 20:345-349, 2006
35. Cook G, Clark RE, Morris TC, et al: A randomized study (WOS MM1) comparing the oral regime Z-Dex (idarubicin and dexamethasone) with vincristine, adriamycin and dexamethasone as induction therapy for newly diagnosed patients with multiple myeloma. Br J Haematol 126:792-798, 2004
36. Corso A, Mangiacavalli S, Barbarano L, et al: Limited feasibility of double transplant in multiple myeloma: results of a multicenter study on 153 patients aged <65 years. Cancer 109:2273-2278, 2007
37. Davies FE, Forsyth PD, Rawstron AC, et al: The impact of attaining a minimal disease state after high-dose melphalan and autologous transplantation for multiple myeloma. Br J Haematol 112:814-819, 2001
38. Desikan KR, Tricot G, Dhodapkar M, et al: Melphalan plus total body irradiation (MEL-TBI) or cyclophosphamide (MEL-CY) as a conditioning regimen with second autotransplant in responding patients with myeloma is inferior compared to historical controls receiving tandem transplants with melphalan alone. Bone Marrow Transplant 25:483-487, 2000
39. Dimopoulos MA, Zervas K, Kouvatseas G, et al: Thalidomide and dexamethasone combination for refractory multiple myeloma. Ann Oncol 12:991-995, 2001
40. Dimopoulos MA, Hamilos G, Zomas A, et al: Pulsed cyclophosphamide, thalidomide and dexamethasone: an oral regimen for previously treated patients with multiple myeloma. Hematol J 5:112-117, 2004
41. Dimopoulos M, Spencer A, Attal M, et al: Lenalidomide plus dexamethasone for relapsed or refractory multiple myeloma. N Engl J Med 357:2123-2132, 2007
42. Dingli D, Rajkumar SV, Nowakowski GS, et al: Combination therapy with thalidomide and dexamethasone in patients with newly diagnosed multiple myeloma not undergoing upfront autologous stem cell transplantation: a phase II trial. Haematologica 90:1650-1654, 2005
43. Dispenzieri, A., S. Jacobus, D.H. Vesole, et al., Primary therapy with single agent bortezomib as induction, maintenance and re-induction in patients with high-risk myeloma: results of the ECOG E2A02 trial. Leukemia, 2010. 24(8): p. 1406-11.
44. Drayson MT, Chapman CE, Dunn JA, et al: MRC trial of alpha2b-interferon maintenance therapy in first plateau phase of multiple myeloma. MRC Working Party on Leukaemia in Adults. Br J Haematol 101:195-202, 1998
45. Dumontet C, Jaubert J, Sebban C, et al: Clinical and pharmacokinetic phase II study of fotemustine in refractory and relapsing multiple myeloma patients. Ann Oncol 14:615-622, 2003
46. Einsele H, Bamberg M, Budach W, et al: A new conditioning regimen involving total marrow irradiation, busulfan and cyclophosphamide followed by autologous PBSCT in patients with advanced multiple myeloma. Bone Marrow Transplant 32:593-599, 2003
47. Eom KS, [Min CK](http://www.ncbi.nlm.nih.gov/pubmed?term=%22Min%20CK%22%5BAuthor%5D), [Lee S](http://www.ncbi.nlm.nih.gov/pubmed?term=%22Lee%20S%22%5BAuthor%5D), et al. Efficacy of up-front treatment with a double stem cell transplantation in multiple myeloma. Jpn J Clin Oncol: 36:432-438, 2006
48. Facon T, [Mary JY](http://www.ncbi.nlm.nih.gov/pubmed?term=%22Mary%20JY%22%5BAuthor%5D), [Pégourie B](http://www.ncbi.nlm.nih.gov/pubmed?term=%22P%C3%A9gourie%20B%22%5BAuthor%5D), et al: Dexamethasone-based regimens versus melphalan-prednisone for elderly multiple myeloma patients ineligible for high-dose therapy. Blood 107:1292-1298, 2006
49. Fassas AB, [Spencer T](http://www.ncbi.nlm.nih.gov/pubmed?term=%22Spencer%20T%22%5BAuthor%5D), [Desikan R](http://www.ncbi.nlm.nih.gov/pubmed?term=%22Desikan%20R%22%5BAuthor%5D), et al: Cytotoxic chemotherapy following tandem autotransplants in multiple myeloma patients. Br J Haematol 119:164-168, 2002
50. Fenk R, [Schneider P](http://www.ncbi.nlm.nih.gov/pubmed?term=%22Schneider%20P%22%5BAuthor%5D), [Kropff M](http://www.ncbi.nlm.nih.gov/pubmed?term=%22Kropff%20M%22%5BAuthor%5D), et al: High-dose idarubicin, cyclophosphamide and melphalan as conditioning for autologous stem cell transplantation increases treatment-related mortality in patients with multiple myeloma: results of a randomised study. Br J Haematol 130:588-594, 2005
51. Fermand JP, Chevret S, Ravaud P, et al: High-dose chemoradiotherapy and autologous blood stem cell transplantation in multiple myeloma: results of a phase II trial involving 63 patients. Blood 82:2005-2009, 1993
52. Fermand JP, [Ravaud P](http://www.ncbi.nlm.nih.gov/pubmed?term=%22Ravaud%20P%22%5BAuthor%5D), [Chevret S](http://www.ncbi.nlm.nih.gov/pubmed?term=%22Chevret%20S%22%5BAuthor%5D), et al: High-dose therapy and autologous peripheral blood stem cell transplantation in multiple myeloma: up-front or rescue treatment? Results of a multicenter sequential randomized clinical trial. Blood 92:3131-3136, 1998
53. Fermand JP, Katsahian S, Divine M, et al: High-dose therapy and autologous blood stem-cell transplantation compared with conventional treatment in myeloma patients aged 55 to 65 years: long-term results of a randomized control trial from the Group Myelome-Autogreffe. J Clin Oncol 23:9227-9233, 2005
54. [Fosså A](http://www.ncbi.nlm.nih.gov/pubmed?term=%22Foss%C3%A5%20A%22%5BAuthor%5D), [Muer M](http://www.ncbi.nlm.nih.gov/pubmed?term=%22Muer%20M%22%5BAuthor%5D), [Kasper C](http://www.ncbi.nlm.nih.gov/pubmed?term=%22Kasper%20C%22%5BAuthor%5D), et al: Bolus vincristine and epirubicin with cyclophosphamide and dexamethasone (VECD) as induction and salvage treatment in multiple myeloma. Leukemia 12:422-426, 1998
55. Friedenberg WR, [Rue M](http://www.ncbi.nlm.nih.gov/pubmed?term=%22Rue%20M%22%5BAuthor%5D), [Blood EA](http://www.ncbi.nlm.nih.gov/pubmed?term=%22Blood%20EA%22%5BAuthor%5D), et al: Phase III study of PSC-833 (valspodar) in combination with vincristine, doxorubicin, and dexamethasone (valspodar/VAD) versus VAD alone in patients with recurring or refractory multiple myeloma (E1A95): a trial of the Eastern Cooperative Oncology Group. Cancer 106:830-838, 2006
56. Garcia-Sanz R, [Hernández JM](http://www.ncbi.nlm.nih.gov/pubmed?term=%22Hern%C3%A1ndez%20JM%22%5BAuthor%5D), [Sureda A](http://www.ncbi.nlm.nih.gov/pubmed?term=%22Sureda%20A%22%5BAuthor%5D), et al: Pegylated liposomal doxorubicin, melphalan and prednisone therapy for elderly patients with multiple myeloma. Hematol Oncol 24:205-211, 2006
57. Ghosh, N., X. Ye, A. Ferguson, et al., Bortezomib and thalidomide, a steroid free regimen in newly diagnosed patients with multiple myeloma. Br J Haematol, 2011. 152(5): p. 593-9.
58. Gianni AM, [Tarella C](http://www.ncbi.nlm.nih.gov/pubmed?term=%22Tarella%20C%22%5BAuthor%5D), [Bregni M](http://www.ncbi.nlm.nih.gov/pubmed?term=%22Bregni%20M%22%5BAuthor%5D), et al: High-dose sequential chemoradiotherapy, a widely applicable regimen, confers survival benefit to patients with high-risk multiple myeloma. J Clin Oncol 12:503-509, 1994
59. Giralt S, [Bensinger W](http://www.ncbi.nlm.nih.gov/pubmed?term=%22Bensinger%20W%22%5BAuthor%5D), [Goodman M](http://www.ncbi.nlm.nih.gov/pubmed?term=%22Goodman%20M%22%5BAuthor%5D), et al: 166Ho-DOTMP plus melphalan followed by peripheral blood stem cell transplantation in patients with multiple myeloma: results of two phase 1/2 trials. Blood 102:2684-2691, 2003
60. Gojo I, [Meisenberg B](http://www.ncbi.nlm.nih.gov/pubmed?term=%22Meisenberg%20B%22%5BAuthor%5D), [Guo C](http://www.ncbi.nlm.nih.gov/pubmed?term=%22Guo%20C%22%5BAuthor%5D), et al: Autologous stem cell transplantation followed by consolidation chemotherapy for patients with multiple myeloma. Bone Marrow Transplant 37:65-72, 2006
61. Harousseau JL, [Milpied N](http://www.ncbi.nlm.nih.gov/pubmed?term=%22Milpied%20N%22%5BAuthor%5D), [Laporte JP](http://www.ncbi.nlm.nih.gov/pubmed?term=%22Laporte%20JP%22%5BAuthor%5D), et al: Double-intensive therapy in high-risk multiple myeloma. Blood 79:2827-2833, 1992
62. Harousseau, J.L., M. Attal, H. Avet-Loiseau, et al., Bortezomib plus dexamethasone is superior to vincristine plus doxorubicin plus dexamethasone as induction treatment prior to autologous stem-cell transplantation in newly diagnosed multiple myeloma: results of the IFM 2005-01 phase III trial. J Clin Oncol, 2011. 28(30): p. 4621-9.
63. Hernández JM, García-Sanz R, Golvano E, et al: Randomized comparison of dexamethasone combined with melphalan versus melphalan with prednisone in the treatment of elderly patients with multiple myeloma. Br J Haematol 127:159-164, 2004
64. Hjorth M, Hellquist L, Holmberg E, et al: Initial versus deferred melphalan-prednisone therapy for asymptomatic multiple myeloma stage I - a randomized study. Myeloma Group of Western Sweden. Eur J Haematol 50:95-102, 1993
65. Hjorth M, Westin J, Dahl IM, et al: Interferon-alpha 2b added to melphalan-prednisone for initial and maintenance therapy in multiple myeloma. A randomized, controlled trial. The Nordic Myeloma Study Group. Ann Intern Med 124:212-222, 1996
66. Horvath N, [Hahn U](http://www.ncbi.nlm.nih.gov/pubmed?term=%22Hahn%20U%22%5BAuthor%5D), [Joshua D](http://www.ncbi.nlm.nih.gov/pubmed?term=%22Joshua%20D%22%5BAuthor%5D), et al: Long-term follow up of sequential mobilisation and autologous transplantation with CD34-selected cells in multiple myeloma: a multimodality approach. Intern Med J 34:167-175, 2004
67. Huijgens PC, [Dekker-Van Roessel HM](http://www.ncbi.nlm.nih.gov/pubmed?term=%22Dekker-Van%20Roessel%20HM%22%5BAuthor%5D), et al: High-dose melphalan with G-CSF-stimulated whole blood rescue followed by stem cell harvesting and busulphan/cyclophosphamide with autologous stem cell transplantation in multiple myeloma. Bone Marrow Transplant 27:925-931, 2001
68. Hulin, C., T. Facon, P. Rodon, et al., Efficacy of melphalan and prednisone plus thalidomide in patients older than 75 years with newly diagnosed multiple myeloma: IFM 01/01 trial. J Clin Oncol, 2009. 27(22): p. 3664-70.
69. Hussein MA, [Wood L](http://www.ncbi.nlm.nih.gov/pubmed?term=%22Wood%20L%22%5BAuthor%5D), [Hsi E](http://www.ncbi.nlm.nih.gov/pubmed?term=%22Hsi%20E%22%5BAuthor%5D), et al: A Phase II trial of pegylated liposomal doxorubicin, vincristine, and reduced-dose dexamethasone combination therapy in newly diagnosed multiple myeloma patients. Cancer 95:2160-2168, 2002
70. Kars A, Celik I, Kansu E, et al: Maintenance therapy with alpha-interferon following first-line VAD in multiple myeloma. Eur J Haematol 59:100-104, 1997
71. Keldsen N, [Bjerrum OW](http://www.ncbi.nlm.nih.gov/pubmed?term=%22Bjerrum%20OW%22%5BAuthor%5D), [Dahl IM](http://www.ncbi.nlm.nih.gov/pubmed?term=%22Dahl%20IM%22%5BAuthor%5D), et al: Multiple myeloma treated with mitoxantrone in combination with vincristine and prednisolone (NOP regimen) versus melphalan and prednisolone: a phase III study. Nordic Myeloma Study Group (NMSG). Eur J Haematol 51:80-85, 1993
72. Kneppers, E., H.M. Lokhorst, C.M. Eeltink, et al., Analysis of efficacy and prognostic factors of lenalidomide treatment as part of a Dutch compassionate use program. Clin Lymphoma Myeloma Leuk, 2011. 10(2): p. 138-43.
73. Kropff MH, [Lang N](http://www.ncbi.nlm.nih.gov/pubmed?term=%22Lang%20N%22%5BAuthor%5D), [Bisping G](http://www.ncbi.nlm.nih.gov/pubmed?term=%22Bisping%20G%22%5BAuthor%5D), et al: Hyperfractionated cyclophosphamide in combination with pulsed dexamethasone and thalidomide (HyperCDT) in primary refractory or relapsed multiple myeloma. Br J Haematol 122:607-616, 2003
74. Kropff M, [Bisping G](http://www.ncbi.nlm.nih.gov/pubmed?term=%22Bisping%20G%22%5BAuthor%5D), [Schuck E](http://www.ncbi.nlm.nih.gov/pubmed?term=%22Schuck%20E%22%5BAuthor%5D), et al: Bortezomib in combination with intermediate-dose dexamethasone and continuous low-dose oral cyclophosphamide for relapsed multiple myeloma. Br J Haematol 138:330-337, 2007
75. Kumar S, Gertz MA, Dispenzieri A, et al: Response rate, durability of response, and survival after thalidomide therapy for relapsed multiple myeloma. Mayo Clin Proc 78:34-39, 2003a
76. Kumar L, Raju GM, Ganessan K, et al: High dose chemotherapy followed by autologous haemopoietic stem cell transplant in multiple myeloma. Natl Med J India 16:16-21, 2003b
77. Kyle, R.A., S. Jacobus, W.R. Friedenberg, et al., The treatment of multiple myeloma using vincristine, carmustine, melphalan, cyclophosphamide, and prednisone (VBMCP) alternating with high-dose cyclophosphamide and alpha(2)beta interferon versus VBMCP: results of a phase III Eastern Cooperative Oncology Group Study E5A93. Cancer, 2009. 115(10): p. 2155-64.
78. Kyriakou C, [Thomson K](http://www.ncbi.nlm.nih.gov/pubmed?term=%22Thomson%20K%22%5BAuthor%5D), [D'Sa S](http://www.ncbi.nlm.nih.gov/pubmed?term=%22D'Sa%20S%22%5BAuthor%5D), et al: Low-dose thalidomide in combination with oral weekly cyclophosphamide and pulsed dexamethasone is a well tolerated and effective regimen in patients with relapsed and refractory multiple myeloma. Br J Haematol 129:763-770, 2005
79. Lacy MQ, [Gertz MA](http://www.ncbi.nlm.nih.gov/pubmed?term=%22Gertz%20MA%22%5BAuthor%5D), [Dispenzieri A](http://www.ncbi.nlm.nih.gov/pubmed?term=%22Dispenzieri%20A%22%5BAuthor%5D), et al: Long-term results of response to therapy, time to progression, and survival with lenalidomide plus dexamethasone in newly diagnosed myeloma. Mayo Clin Proc 82:1179-1184, 2007
80. Lahuerta JJ, Martinez-Lopez J, Grande C, et al: Conditioning regimens in autologous stem cell transplantation for multiple myeloma: a comparative study of efficacy and toxicity from the Spanish Registry for Transplantation in Multiple Myeloma. Br J Haematol 109:138-147, 2000
81. Lahuerta JJ, [Grande C](http://www.ncbi.nlm.nih.gov/pubmed?term=%22Grande%20C%22%5BAuthor%5D), [Martínez-Lopez J](http://www.ncbi.nlm.nih.gov/pubmed?term=%22Mart%C3%ADnez-Lopez%20J%22%5BAuthor%5D), et al: Tandem transplants with different high-dose regimens improve the complete remission rates in multiple myeloma. Results of a Grupo Español de Síndromes Linfoproliferativos/Trasplante Autólogo de Médula Osea phase II trial. Br J Haematol 120:296-303, 2003
82. Lee JL, Kim SB, Lee GW, et al: The efficacy of high-dose melphalan with autologous peripheral blood stem cell transplantation in patients with multiple myeloma. Yonsei Med J 44:800-810, 2003
83. Lee, S.S., C. Suh, B.S. Kim, et al., Bortezomib, doxorubicin, and dexamethasone combination therapy followed by thalidomide and dexamethasone consolidation as a salvage treatment for relapsed or refractory multiple myeloma: analysis of efficacy and safety. Ann Hematol, 2010. 89(9): p. 905-12.
84. Lenhoff S, [Hjorth M](http://www.ncbi.nlm.nih.gov/pubmed?term=%22Hjorth%20M%22%5BAuthor%5D), [Turesson I](http://www.ncbi.nlm.nih.gov/pubmed?term=%22Turesson%20I%22%5BAuthor%5D), et al: Intensive therapy for multiple myeloma in patients younger than 60 years. Long-term results focusing on the effect of the degree of response on survival and relapse pattern after transplantation. Haematologica 91:1228-1233, 2006
85. Lokhorst, H.M., B. van der Holt, S. Zweegman, et al., A randomized phase 3 study on the effect of thalidomide combined with adriamycin, dexamethasone, and high-dose melphalan, followed by thalidomide maintenance in patients with multiple myeloma. Blood, 2010. 115(6): p. 1113-20
86. Ludwig H, Cohen AM, Polliack A, et al: Interferon-alpha for induction and maintenance in multiple myeloma: results of two multicenter randomized trials and summary of other studies. Ann Oncol 6:467-476, 1995
87. Ludwig H, Spicka I, Klener P, et al: Continuous prednisolone versus conventional prednisolone with VMCP-interferon-alpha 2b as first-line chemotherapy in elderly patients with multiple myeloma. Br J Haematol 131:329-337, 2005
88. Ludwig, H., Z. Adam, E. Tothova, et al., Thalidomide maintenance treatment increases progression-free but not overall survival in elderly patients with myeloma. Haematologica, 2010. 95(9): p. 1548-54
89. 13. Ludwig, H., R. Hajek, E. Tothova, et al., Thalidomide-dexamethasone compared with melphalan-prednisolone in elderly patients with multiple myeloma. Blood, 2009. 113(15): p. 3435-42
90. Martino M, Console G, Callea V, et al: Low tolerance and high toxicity of thalidomide as maintenance therapy after double autologous stem cell transplant in multiple myeloma patients. Eur J Haematol 78:35-40, 2007
91. Mellqvist, U.H., S. Lenhoff, H.E. Johnsen, et al., Cyclophosphamide plus dexamethasone is an efficient initial treatment before high-dose melphalan and autologous stem cell transplantation in patients with newly diagnosed multiple myeloma: results of a randomized comparison with vincristine, doxorubicin, and dexamethasone. Cancer, 2008. 112(1): p. 129-35
92. Mileshkin L, Biagi JJ, Mitchell P, et al: Multicenter phase 2 trial of thalidomide in relapsed/refractory multiple myeloma: adverse prognostic impact of advanced age. Blood 102:69-77, 2003
93. Moreau P, [Facon T](http://www.ncbi.nlm.nih.gov/pubmed?term=%22Facon%20T%22%5BAuthor%5D), [Attal M](http://www.ncbi.nlm.nih.gov/pubmed?term=%22Attal%20M%22%5BAuthor%5D), et al: Comparison of 200 mg/m^2^ melphalan and 8 Gy total body irradiation plus 140 mg/m^2^ melphalan as conditioning regimens for peripheral blood stem cell transplantation in patients with newly diagnosed multiple myeloma: final analysis of the Intergroupe Francophone du Myélome 9502 randomized trial. Blood 99:731-735, 2002
94. Moreau P, [Hullin C](http://www.ncbi.nlm.nih.gov/pubmed?term=%22Hullin%20C%22%5BAuthor%5D), [Garban F](http://www.ncbi.nlm.nih.gov/pubmed?term=%22Garban%20F%22%5BAuthor%5D), et al: Tandem autologous stem cell transplantation in high-risk de novo multiple myeloma: final results of the prospective and randomized IFM 99-04 protocol. Blood 107:397-403, 2006
95. Offidani M, Olivieri A, Montillo M, et al: Two dosage interferon-alpha 2b maintenance therapy in patients affected by low-risk multiple myeloma in plateau phase: a randomized trial. Haematologica 83:40-47, 1998
96. Offidani M, [Mele A](http://www.ncbi.nlm.nih.gov/pubmed?term=%22Mele%20A%22%5BAuthor%5D), [Corvatta L](http://www.ncbi.nlm.nih.gov/pubmed?term=%22Corvatta%20L%22%5BAuthor%5D), et al: Gemcitabine alone or combined with cisplatin in relapsed or refractory multiple myeloma. Leuk Lymphoma 43:1273-1279, 2002
97. Offidani M, [Corvatta L](http://www.ncbi.nlm.nih.gov/pubmed?term=%22Corvatta%20L%22%5BAuthor%5D), [Marconi M](http://www.ncbi.nlm.nih.gov/pubmed?term=%22Marconi%20M%22%5BAuthor%5D), et al: Thalidomide plus oral melphalan compared with thalidomide alone for advanced multiple myeloma. Hematol J 5:312-317, 2004
98. Offidani M, [Corvatta L](http://www.ncbi.nlm.nih.gov/pubmed?term=%22Corvatta%20L%22%5BAuthor%5D), [Marconi M](http://www.ncbi.nlm.nih.gov/pubmed?term=%22Marconi%20M%22%5BAuthor%5D), et al: Low-dose thalidomide with pegylated liposomal doxorubicin and high-dose dexamethasone for relapsed/refractory multiple myeloma: a prospective, multicenter, phase II study. Haematologica 91:133-136, 2006
99. Offidani, M., L. Corvatta, C. Polloni, et al., Thalidomide-dexamethasone versus interferon-alpha-dexamethasone as maintenance treatment after ThaDD induction for multiple myeloma: a prospective, multicentre, randomised study. Br J Haematol, 2009. 144(5): p. 653-9
100. Orlowski RZ, [Nagler A](http://www.ncbi.nlm.nih.gov/pubmed?term=%22Nagler%20A%22%5BAuthor%5D), [Sonneveld P](http://www.ncbi.nlm.nih.gov/pubmed?term=%22Sonneveld%20P%22%5BAuthor%5D), et al: Randomized phase III study of pegylated liposomal doxorubicin plus bortezomib compared with bortezomib alone in relapsed or refractory multiple myeloma: combination therapy improves time to progression. J Clin Oncol 25:3892-3901, 2007
101. Ossenkoppele GJ, [Schuurhuis GJ](http://www.ncbi.nlm.nih.gov/pubmed?term=%22Schuurhuis%20GJ%22%5BAuthor%5D), [Jonkhoff AR](http://www.ncbi.nlm.nih.gov/pubmed?term=%22Jonkhoff%20AR%22%5BAuthor%5D), et al: High-dose melphalan with re-infusion of unprocessed, G-CSF-primed whole blood is effective and non-toxic therapy in multiple myeloma. Eur J Cancer 32A:2058-2063, 1996
102. Palumbo A, [Boccadoro M](http://www.ncbi.nlm.nih.gov/pubmed?term=%22Boccadoro%20M%22%5BAuthor%5D), [Garino LA](http://www.ncbi.nlm.nih.gov/pubmed?term=%22Garino%20LA%22%5BAuthor%5D), et al: Interferon plus glucocorticoids as intensified maintenance therapy prolongs tumor control in relapsed myeloma. Acta Haematol 90:71-76, 1993
103. Palumbo A, [Triolo S](http://www.ncbi.nlm.nih.gov/pubmed?term=%22Triolo%20S%22%5BAuthor%5D), [Argentino C](http://www.ncbi.nlm.nih.gov/pubmed?term=%22Argentino%20C%22%5BAuthor%5D), et al: Dose-intensive melphalan with stem cell support (MEL100) is superior to standard treatment in elderly myeloma patients. Blood 94:1248-1253, 1999
104. Palumbo A, Bertola A, Falco P, et al: Efficacy of low-dose thalidomide and dexamethasone as first salvage regimen in multiple myeloma. Hematol J 5:318-324, 2004a
105. Palumbo A, [Bringhen S](http://www.ncbi.nlm.nih.gov/pubmed?term=%22Bringhen%20S%22%5BAuthor%5D), [Petrucci MT](http://www.ncbi.nlm.nih.gov/pubmed?term=%22Petrucci%20MT%22%5BAuthor%5D), et al: Intermediate-dose melphalan improves survival of myeloma patients aged 50 to 70: results of a randomized controlled trial. Blood 104:3052-3057, 2004b
106. Palumbo A, [Bertola A](http://www.ncbi.nlm.nih.gov/pubmed?term=%22Bertola%20A%22%5BAuthor%5D), [Musto P](http://www.ncbi.nlm.nih.gov/pubmed?term=%22Musto%20P%22%5BAuthor%5D), et al: Oral melphalan, prednisone, and thalidomide for newly diagnosed patients with myeloma. Cancer 104:1428-1433, 2005
107. Palumbo A, Avonto I, Bruno B, et al: Intravenous melphalan, thalidomide and prednisone in refractory and relapsed multiple myeloma. Eur J Haematol 76:273-277, 2006a
108. Palumbo A, [Bringhen S](http://www.ncbi.nlm.nih.gov/pubmed?term=%22Bringhen%20S%22%5BAuthor%5D), [Caravita T](http://www.ncbi.nlm.nih.gov/pubmed?term=%22Caravita%20T%22%5BAuthor%5D), et al: Oral melphalan and prednisone chemotherapy plus thalidomide compared with melphalan and prednisone alone in elderly patients with multiple myeloma: randomised controlled trial. Lancet 367:825-831, 2006b
109. Palumbo A, [Ambrosini MT](http://www.ncbi.nlm.nih.gov/pubmed?term=%22Ambrosini%20MT%22%5BAuthor%5D), [Benevolo G](http://www.ncbi.nlm.nih.gov/pubmed?term=%22Benevolo%20G%22%5BAuthor%5D), et al: Bortezomib, melphalan, prednisone, and thalidomide for relapsed multiple myeloma. Blood 109:2767-2772, 2007
110. Palumbo, A., F. Gay, S. Bringhen, et al., Bortezomib, doxorubicin and dexamethasone in advanced multiple myeloma. Ann Oncol, 2008. 19(6): p. 1160-5
111. Palumbo, A., A. Larocca, P. Falco, et al., Lenalidomide, melphalan, prednisone and thalidomide (RMPT) for relapsed/refractory multiple myeloma. Leukemia, 2010. 24(5): p. 1037-42
112. Peest D, [Deicher H](http://www.ncbi.nlm.nih.gov/pubmed?term=%22Deicher%20H%22%5BAuthor%5D), [Coldewey R](http://www.ncbi.nlm.nih.gov/pubmed?term=%22Coldewey%20R%22%5BAuthor%5D), et al: A comparison of polychemotherapy and melphalan/prednisone for primary remission induction, and interferon-alpha for maintenance treatment, in multiple myeloma. A prospective trial of the German Myeloma Treatment Group. Eur J Cancer 31A:146-151, 1995
113. Pönisch W, Mitrou PS, Merkle K, et al: Treatment of bendamustine and prednisone in patients with newly diagnosed multiple myeloma results in superior complete response rate, prolonged time to treatment failure and improved quality of life compared to treatment with melphalan and prednisone - a randomized phase III study of the East German Study Group of Hematology and Oncology (OSHO). J Cancer Res Clin Oncol 132:205-212, 2006
114. Powles R, [Sirohi B](http://www.ncbi.nlm.nih.gov/pubmed?term=%22Sirohi%20B%22%5BAuthor%5D), [Kulkarni S](http://www.ncbi.nlm.nih.gov/pubmed?term=%22Kulkarni%20S%22%5BAuthor%5D), et al: Acute lymphoblastic leukaemia-type intensive chemotherapy to eliminate minimal residual disease after high-dose melphalan and autologous transplantation in multiple myeloma - a phase I/II feasibility and tolerance study of 17 patients. Bone Marrow Transplant 25:949-956, 2000
115. Prince HM, [Mileshkin L](http://www.ncbi.nlm.nih.gov/pubmed?term=%22Mileshkin%20L%22%5BAuthor%5D), [Roberts A](http://www.ncbi.nlm.nih.gov/pubmed?term=%22Roberts%20A%22%5BAuthor%5D), et al: A multicenter phase II trial of thalidomide and celecoxib for patients with relapsed and refractory multiple myeloma. Clin Cancer Res 11:5504-5514. 2005
116. Putkonen M, [Rauhala A](http://www.ncbi.nlm.nih.gov/pubmed?term=%22Rauhala%20A%22%5BAuthor%5D), [Itälä M](http://www.ncbi.nlm.nih.gov/pubmed?term=%22It%C3%A4l%C3%A4%20M%22%5BAuthor%5D), et al: Double versus single autotransplantation in multiple myeloma; a single center experience of 100 patients. Haematologica 90:562-563, 2005
117. Rajkumar, S.V., S. Jacobus, N.S. Callander, et al., Lenalidomide plus high-dose dexamethasone versus lenalidomide plus low-dose dexamethasone as initial therapy for newly diagnosed multiple myeloma: an open-label randomised controlled trial. Lancet Oncol, 2010. 11(1): p. 29-37
118. Rajkumar, S.V., L. Rosinol, M. Hussein, et al., Multicenter, randomized, double-blind, placebo-controlled study of thalidomide plus dexamethasone compared with dexamethasone as initial therapy for newly diagnosed multiple myeloma. J Clin Oncol, 2008. 26(13): p. 2171-7
119. Remes K, Itälä M, Kauppila M, et al: Autologous blood cell transplantation in multiple myeloma: impact of CD34+ cell selection with long follow-up. J Hematother Stem Cell Res 12:63-70, 2003
120. Reynolds C, Ratanatharathorn V, Adams P, et al: Allogeneic stem cell transplantation reduces disease progression compared to autologous transplantation in patients with multiple myeloma. Bone Marrow Transplant 27:801-807, 2001
121. Ria R, [Falzetti F](http://www.ncbi.nlm.nih.gov/pubmed?term=%22Falzetti%20F%22%5BAuthor%5D), [Ballanti S](http://www.ncbi.nlm.nih.gov/pubmed?term=%22Ballanti%20S%22%5BAuthor%5D), et al: Melphalan versus melphalan plus busulphan in conditioning to autologous stem cell transplantation for low-risk multiple myeloma. Hematol J 5:118-122, 2004
122. Richardson PG, [Barlogie B](http://www.ncbi.nlm.nih.gov/pubmed?term=%22Barlogie%20B%22%5BAuthor%5D), [Berenson J](http://www.ncbi.nlm.nih.gov/pubmed?term=%22Berenson%20J%22%5BAuthor%5D), et al: A phase 2 study of bortezomib in relapsed, refractory myeloma. N Engl J Med 348:2609-2617, 2003
123. Richardson P, [Schlossman R](http://www.ncbi.nlm.nih.gov/pubmed?term=%22Schlossman%20R%22%5BAuthor%5D), [Jagannath S](http://www.ncbi.nlm.nih.gov/pubmed?term=%22Jagannath%20S%22%5BAuthor%5D), et al: Thalidomide for patients with relapsed multiple myeloma after high-dose chemotherapy and stem cell transplantation: results of an open-label multicenter phase 2 study of efficacy, toxicity, and biological activity. Mayo Clin Proc 79:875-882, 2004
124. Richardson PG, Barlogie B, Berenson J, et al: Extended follow-up of a phase II trial in relapsed, refractory multiple myeloma: final time-to-event results from the SUMMIT trial. Cancer 106:1316-1319, 2006a
125. Richardson PG, [Blood E](http://www.ncbi.nlm.nih.gov/pubmed?term=%22Blood%20E%22%5BAuthor%5D), [Mitsiades CS](http://www.ncbi.nlm.nih.gov/pubmed?term=%22Mitsiades%20CS%22%5BAuthor%5D), et al: A randomized phase 2 study of lenalidomide therapy for patients with relapsed or relapsed and refractory multiple myeloma. Blood 108:3458-3464, 2006b
126. Richardson PG, [Sonneveld P](http://www.ncbi.nlm.nih.gov/pubmed?term=%22Sonneveld%20P%22%5BAuthor%5D), [Schuster M](http://www.ncbi.nlm.nih.gov/pubmed?term=%22Schuster%20M%22%5BAuthor%5D), et al: Extended follow-up of a phase 3 trial in relapsed multiple myeloma: final time-to-event results of the APEX trial. Blood 110:3557-3560, 2007
127. Richardson, P., S. Jagannath, M. Hussein, et al., Safety and efficacy of single-agent lenalidomide in patients with relapsed and refractory multiple myeloma. Blood, 2009. 114(4): p. 772-8
128. Rifkin RM, [Gregory SA](http://www.ncbi.nlm.nih.gov/pubmed?term=%22Gregory%20SA%22%5BAuthor%5D), [Mohrbacher A](http://www.ncbi.nlm.nih.gov/pubmed?term=%22Mohrbacher%20A%22%5BAuthor%5D), et al: Pegylated liposomal doxorubicin, vincristine, and dexamethasone provide significant reduction in toxicity compared with doxorubicin, vincristine, and dexamethasone in patients with newly diagnosed multiple myeloma: a Phase III multicenter randomized trial. Cancer 106:848-858, 2006
129. Sahebi F, [Spielberger R](http://www.ncbi.nlm.nih.gov/pubmed?term=%22Spielberger%20R%22%5BAuthor%5D), [Kogut NM](http://www.ncbi.nlm.nih.gov/pubmed?term=%22Kogut%20NM%22%5BAuthor%5D), et al: Maintenance thalidomide following single cycle autologous peripheral blood stem cell transplant in patients with multiple myeloma. Bone Marrow Transplant 37:825-829, 2006
130. Salmon SE, [Crowley JJ](http://www.ncbi.nlm.nih.gov/pubmed?term=%22Crowley%20JJ%22%5BAuthor%5D), [Grogan TM](http://www.ncbi.nlm.nih.gov/pubmed?term=%22Grogan%20TM%22%5BAuthor%5D), et al: Combination chemotherapy, glucocorticoids, and interferon alfa in the treatment of multiple myeloma: a Southwest Oncology Group study. J Clin Oncol 12:2405-2414, 1994
131. Salmon SE, [Crowley JJ](http://www.ncbi.nlm.nih.gov/pubmed?term=%22Crowley%20JJ%22%5BAuthor%5D), [Balcerzak SP](http://www.ncbi.nlm.nih.gov/pubmed?term=%22Balcerzak%20SP%22%5BAuthor%5D), et al: Interferon versus interferon plus prednisone remission maintenance therapy for multiple myeloma: a Southwest Oncology Group Study. J Clin Oncol 16:890-896, 1998
132. San Miguel, J.F., R. Schlag, N.K. Khuageva, et al., Bortezomib plus melphalan and prednisone for initial treatment of multiple myeloma. N Engl J Med, 2008. 359(9): p. 906-17
133. Schaar CG, [Kluin-Nelemans HC](http://www.ncbi.nlm.nih.gov/pubmed?term=%22Kluin-Nelemans%20HC%22%5BAuthor%5D), [Te Marvelde C](http://www.ncbi.nlm.nih.gov/pubmed?term=%22Te%20Marvelde%20C%22%5BAuthor%5D), et al: Interferon-alpha as maintenance therapy in patients with multiple myeloma. Ann Oncol 16:634-639, 2005
134. Schey SA, [Cavenagh J](http://www.ncbi.nlm.nih.gov/pubmed?term=%22Cavenagh%20J%22%5BAuthor%5D), [Johnson R](http://www.ncbi.nlm.nih.gov/pubmed?term=%22Johnson%20R%22%5BAuthor%5D), et al: An UK myeloma forum phase II study of thalidomide; long term follow-up and recommendations for treatment. Leuk Res 27:909-914, 2003
135. Segeren CM, [Sonneveld P](http://www.ncbi.nlm.nih.gov/pubmed?term=%22Sonneveld%20P%22%5BAuthor%5D), [van der Holt B](http://www.ncbi.nlm.nih.gov/pubmed?term=%22van%20der%20Holt%20B%22%5BAuthor%5D), et al: Overall and event-free survival are not improved by the use of myeloablative therapy following intensified chemotherapy in previously untreated patients with multiple myeloma: a prospective randomized phase 3 study. Blood 101:2144-2151, 2003
136. Sharma A, Lokeshwar N, Raina V, et al: Melphalan and dexamethasone for patients with multiple myeloma who are not candidates for autologous stem cell transplantation. Natl Med J India 20:121-124, 2007
137. Shustik C, [Belch A](http://www.ncbi.nlm.nih.gov/pubmed?term=%22Belch%20A%22%5BAuthor%5D), [Robinson S](http://www.ncbi.nlm.nih.gov/pubmed?term=%22Robinson%20S%22%5BAuthor%5D), et al: A randomised comparison of melphalan with prednisone or dexamethasone as induction therapy and dexamethasone or observation as maintenance therapy in multiple myeloma: NCIC CTG MY.7. Br J Haematol 136:203-211, 2006
138. Singhal S, [Mehta J](http://www.ncbi.nlm.nih.gov/pubmed?term=%22Mehta%20J%22%5BAuthor%5D), [Desikan R](http://www.ncbi.nlm.nih.gov/pubmed?term=%22Desikan%20R%22%5BAuthor%5D), et al: Antitumor activity of thalidomide in refractory multiple myeloma. N Engl J Med 341:1565-1571, 1999
139. Sonneveld P, Suciu S, Weijermans P, et al: Cyclosporin A combined with vincristine, doxorubicin and dexamethasone (VAD) compared with VAD alone in patients with advanced refractory multiple myeloma: an EORTC-HOVON randomized phase III study (06914). Br J Haematol 115:895-902, 2001
140. Sonneveld P, [van der Holt B](http://www.ncbi.nlm.nih.gov/pubmed?term=%22van%20der%20Holt%20B%22%5BAuthor%5D), [Segeren CM](http://www.ncbi.nlm.nih.gov/pubmed?term=%22Segeren%20CM%22%5BAuthor%5D), et al: Intermediate-dose melphalan compared with myeloablative treatment in multiple myeloma: long-term follow-up of the Dutch Cooperative Group HOVON 24 trial. Haematologica 92:928-935, 2007
141. Spencer A, [Seldon M](http://www.ncbi.nlm.nih.gov/pubmed?term=%22Seldon%20M%22%5BAuthor%5D), [Deveridge S](http://www.ncbi.nlm.nih.gov/pubmed?term=%22Deveridge%20S%22%5BAuthor%5D), et al: Induction with oral chemotherapy (CID) followed by early autologous stem cell transplantation for de novo multiple myeloma patients. Hematol J 5:216-221, 2004
142. Stewart AK, [Vescio R](http://www.ncbi.nlm.nih.gov/pubmed?term=%22Vescio%20R%22%5BAuthor%5D), [Schiller G](http://www.ncbi.nlm.nih.gov/pubmed?term=%22Schiller%20G%22%5BAuthor%5D), et al: Purging of autologous peripheral-blood stem cells using CD34 selection does not improve overall or progression-free survival after high-dose chemotherapy for multiple myeloma: results of a multicenter randomized controlled trial. J Clin Oncol 19:3771-3779, 2001
143. Stewart AK, [Chen CI](http://www.ncbi.nlm.nih.gov/pubmed?term=%22Chen%20CI%22%5BAuthor%5D), [Howson-Jan K](http://www.ncbi.nlm.nih.gov/pubmed?term=%22Howson-Jan%20K%22%5BAuthor%5D), et al: Results of a multicenter randomized phase II trial of thalidomide and prednisone maintenance therapy for multiple myeloma after autologous stem cell transplant. Clin Cancer Res 10:8170-8176, 2004
144. Suvannasankha A, [Smith GG](http://www.ncbi.nlm.nih.gov/pubmed?term=%22Smith%20GG%22%5BAuthor%5D), [Juliar BE](http://www.ncbi.nlm.nih.gov/pubmed?term=%22Juliar%20BE%22%5BAuthor%5D), et al: Weekly bortezomib/methylprednisolone is effective and well tolerated in relapsed multiple myeloma. Clin Lymphoma Myeloma 7:131-134, 2006
145. Takenaka T, Itoh K, Suzuki T, et al: Phase III study of ranimustine, cyclophosphamide, vincristine, melphalan, and prednisolone (MCNU-COP/MP) versus modified COP/MP in multiple myeloma: a Japan clinical oncology group study, JCOG 9301. Int J Hematol 79:165-173, 2004
146. Vesole DH, [Crowley JJ](http://www.ncbi.nlm.nih.gov/pubmed?term=%22Crowley%20JJ%22%5BAuthor%5D), [Catchatourian R](http://www.ncbi.nlm.nih.gov/pubmed?term=%22Catchatourian%20R%22%5BAuthor%5D), et al: High-dose melphalan with autotransplantation for refractory multiple myeloma: results of a Southwest Oncology Group phase II trial. J Clin Oncol 17:2173-2179, 1999
147. Waage, A., P. Gimsing, P. Fayers, et al., Melphalan and prednisone plus thalidomide or placebo in elderly patients with multiple myeloma. Blood, 2010. 116(9): p. 1405-12
148. Weber DM, [Chen C](http://www.ncbi.nlm.nih.gov/pubmed?term=%22Chen%20C%22%5BAuthor%5D), [Niesvizky R](http://www.ncbi.nlm.nih.gov/pubmed?term=%22Niesvizky%20R%22%5BAuthor%5D), et al: Lenalidomide plus dexamethasone for relapsed multiple myeloma in North America. N Engl J Med 357:2133-2142, 2007
149. Wijermans, P., M. Schaafsma, F. Termorshuizen, et al., Phase III study of the value of thalidomide added to melphalan plus prednisone in elderly patients with newly diagnosed multiple myeloma: the HOVON 49 Study. J Clin Oncol, 2010. 28(19): p. 3160-6
150. Yakoub-Agha I, [Attal M](http://www.ncbi.nlm.nih.gov/pubmed?term=%22Attal%20M%22%5BAuthor%5D), [Dumontet C](http://www.ncbi.nlm.nih.gov/pubmed?term=%22Dumontet%20C%22%5BAuthor%5D), et al: Thalidomide in patients with advanced multiple myeloma: a study of 83 patients-report of the Intergroupe Francophone du Myélome (IFM). Hematol J 3:185-192, 2002
151. Zervas K, [Mihou D](http://www.ncbi.nlm.nih.gov/pubmed?term=%22Mihou%20D%22%5BAuthor%5D), [Katodritou E](http://www.ncbi.nlm.nih.gov/pubmed?term=%22Katodritou%20E%22%5BAuthor%5D), et al: VAD-doxil versus VAD-doxil plus thalidomide as initial treatment for multiple myeloma: results of a multicenter randomized trial of the Greek Myeloma Study Group. Ann Oncol 18:1369-1375, 2007
152. Zonder, J.A., J. Crowley, M.A. Hussein, et al., Lenalidomide and high-dose dexamethasone compared with dexamethasone as initial therapy for multiple myeloma: a randomized Southwest Oncology Group trial (S0232). Blood, 2010. 116(26): p. 5838-41
